# Current Insights into the Metabolome during Hypothermic Kidney Perfusion—A Scoping Review

**DOI:** 10.3390/jcm12113613

**Published:** 2023-05-23

**Authors:** Laurence Verstraeten, Rutger Den abt, Bart Ghesquière, Ina Jochmans

**Affiliations:** 1 Laboratory of Abdominal Transplantation KU Leuven, Transplantation Research Group, Department of Microbiology, Immunology and Transplantation, KU Leuven, 3000 Leuven, Belgium; 2 Metabolomics Expertise Center, Center for Cancer Biology, VIB, 3000 Leuven, Belgium; 3 Laboratory of Applied Mass Spectrometry, Department of Cellular and Molecular Medicine, KU Leuven, 3000 Leuven, Belgium; 4 Department of Abdominal Transplant Surgery, University Hospitals Leuven, 3000 Leuven, Belgium

**Keywords:** hypothermic kidney perfusion, isolated organ perfusion, kidney preservation, kidney metabolism, isotopic tracer, scoping review

## Abstract

This scoping review summarizes what is known about kidney metabolism during hypothermic perfusion preservation. Papers studying kidney metabolism during hypothermic (<12 °C) perfusion were identified (PubMed, Embase, Web of Science, Cochrane). Out of 14,335 initially identified records, 52 were included [dog (26/52), rabbit (2/52), pig (20/52), human (7/52)]. These were published between 1970–2023, partially explaining study heterogeneity. There is a considerable risk of bias in the reported studies. Studies used different perfusates, oxygenation levels, kidney injury levels, and devices and reported on perfusate and tissue metabolites. In 11 papers, (non)radioactively labeled metabolites (tracers) were used to study metabolic pathways. Together these studies show that kidneys are metabolically active during hypothermic perfusion, regardless of the perfusion setting. Although tracers give us more insight into active metabolic pathways, kidney metabolism during hypothermic perfusion is incompletely understood. Metabolism is influenced by perfusate composition, oxygenation levels, and likely also by pre-existing ischemic injury. In the modern era, with increasing donations after circulatory death and the emergence of hypothermic oxygenated perfusion, the focus should be on understanding metabolic perturbations caused by pre-existing injury levels and the effect of perfusate oxygen levels. The use of tracers is indispensable to understanding the kidney’s metabolism during perfusion, given the complexity of interactions between different metabolites.

## 1. Introduction

Hypothermic perfusion preservation, also called hypothermic machine perfusion, reduces the risk of delayed graft function [1]. Studies also suggest improved graft survival in kidneys donated after brain death (DBD), though this is not the case for kidneys donated after circulatory death (DCD) [2,3,4]. A recent randomized controlled trial suggests that actively oxygenating the perfusate during hypothermic perfusion of older DCD kidneys improves kidney function and survival [5]. The underlying mechanisms by which hypothermic perfusion exerts its effect are still incompletely understood.

To understand the mechanisms that drive the effect of hypothermic perfusion preservation and how this technique might be further improved, it is essential to understand “on-pump” kidney behavior. A key concept of hypothermic preservation is that metabolism, and therefore cellular metabolic requirements, are minimized. However, although the metabolic rate below 4 °C is reported to be about 5–10% of that at body temperature [6,7], there is still active metabolism in the cold. Furthermore, the preservation temperature during hypothermic perfusion often does not reach below 4 °C. The metabolic activity in this ex situ, hypothermic environment and how it is influenced by oxygenation is poorly understood [5,8,9].

This review assesses, appraises, and summarizes our current understanding of the kidney’s metabolism during hypothermic perfusion preservation.

## 2. Materials and Methods

### 2.1. Search Strategy

This review was conducted in accordance with the Preferred Reporting Items for Systematic Reviews and Meta-Analyses Extension for Scoping Reviews (PRISMA-ScR) guidelines. The protocol was prospectively registered [10]. With the help of an experienced biomedical information specialist, a search strategy was built and PubMed, Embase, Cochrane Library, and Web of Science Core Collection were searched. The following concepts: “metabolism”, “kidney”, and “perfusion” were developed. The complete search strategy can be found in Table S1.

### 2.2. Study Selection and Eligibility Criteria

Two authors independently assessed the eligibility of the articles based on the title and abstract, conducted full-text analysis, and extracted data. In case of disagreements, a third experienced researcher was consulted. Studies were included from database inception with final searches carried out on 2 February 2023.

Studies were eligible for inclusion if they reported on any of the pre-specified outcomes. Only studies in mammals providing data on perfusate or tissue metabolites of kidneys undergoing hypothermic perfusion were included. Table S2 lists the inclusion and exclusion criteria. Articles written in a language other than English, French, or Dutch; articles with no full text available; review articles, letters, editorials, and conference abstracts were also excluded. Reference lists of included studies were also searched using the same inclusion and exclusion criteria (‘snowballing’).

### 2.3. Data Extraction and Processing

The results of the search were imported into Endnote (Version 20, Clearview Analytics, Philadelphia, PA, USA). Duplicates were removed using the “Find duplicates” tool in Endnote. The remaining articles were imported into Rayyan [11] and screened according to prespecified inclusion and exclusion criteria (Table S2). A data extraction table was designed and tested before extraction of data began. The full data extraction table is publicly accessible and contains information on title, authors, year of publication, study type, experimental set-up, group characteristics, perfusion characteristics, analyses, perfusate results, urine results, tissue results, and post-transplant results [12]. If details of the experimental design were not reported, we attempted to derive information from referenced studies.

Kidneys were categorized as “minimally injured” when they endured less than 5 min of warm ischemia and <30 min of cold ischemia and as “injured” in all other cases. Some articles report on multiple experiments that include different species, oxygenation methods, and perfusates. Therefore, percentages do not always add up to 100%.

### 2.4. Quality Assessment and Data Analysis

Concerning experimental animal studies, the ‘systematic review center for laboratory animal experimentation (SYRCLE) risk of bias tool’ was used to assess the quality of the animal experiments and the article. This tool is based on the Cochrane risk-of-bias tool and adapted for aspects of bias that play a specific role in animal intervention studies [13]. Signal questions were formulated by Hooijmans et al. to facilitate judgment and reported to increase transparency and applicability of results [13]. For studies with human organs, methodological quality was assessed using the National Institutes of Health (NIH) scoring tools. These tools include items for evaluating potential flaws in study methods or implementation, including sources of bias (e.g., patient selection, performance, attrition, and detection), confounding, study power, the strength of causality in the association between interventions and outcomes, and other factors [14].

## 3. Results

A systematic search of online databases, performed on 2 February 2023, resulted in the identification of 14,335 records. After duplicate removal, 9794 records remained, of which 9596 were excluded based upon predefined inclusion and exclusion criteria at the time of initial screening and 152 at the time of full-text screening, leaving 46 included articles. From the reference lists, another 1507 potential papers were identified leading to six additional inclusions. In total, 52 papers were included in this scoping review. Figure S1 shows the flowchart. The full data extraction table can be accessed online [12]. Included articles were published between 1970 and 2023 (Table S3).

### 3.1. Quality and Risk of Bias Assessment

For animal studies, the risk of bias was most often “unclear” or “high” because essential information was often not reported (Figure S2, Table S4). For human studies, the overall quality was better, most often “good” or “fair” (Figure S2, Table S5).

### 3.2. Hypothermic Perfusion Set-up

**Figure 1:**
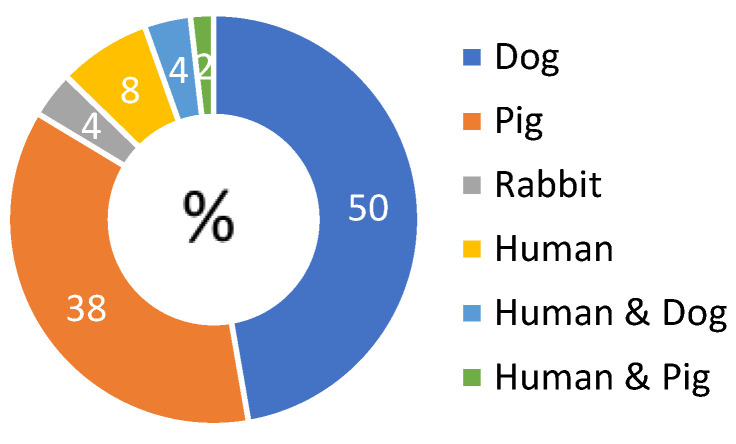
Diagram of distribution of included papers according to the species studied. Some articles report on multiple experiments in different species, therefore percentages do not add up to 100%.

The majority of articles reported on animal experiments (48/52) in dogs (26/52), rabbits (2/52), and pigs (20/52) (Figure 1). Seven studies reported on the perfusion of human kidneys (7/52), of which three were transplant studies [8,15,16]. Some papers report on both animal and human kidney perfusions (3/51) (Table S3). Kidneys were exposed to variable ischemic injury, induced by introducing warm ischemia (5 to 240 min; clamping of the renal artery (and vein) before procurement or procurement after death) or exposure to cold storage before perfusion (up to 20 h) (Table S3). After hypothermic perfusion, kidneys were transplanted in 22 studies [8,15,16,17,18,19,20,21,22,23,24,25,26,27,28,29,30,31,32,33,34,35] or re-perfused with a blood-based perfusate in one study [36]. Kidneys were flushed with different solutions (detailed in the extraction table [12]) before mounting on the perfusion device.

Several perfusion devices were used, from homemade to commercially available devices (Table S3). The latter were sometimes adjusted to fit the aim of the research. Common features of these perfusion circuits were a reservoir and tubing, and a pump to circulate the perfusate. Cooling (2–12 °C) was accomplished by a heat exchanger, ice surrounding the reservoir, or a combination of both. The majority of the circuits used pulsatile perfusion (roller, centrifugal, or peristaltic pump); continuous non-pulsatile perfusion was used in two studies [37,38]. In most studies, perfusion was pressure controlled (25 to 60 mmHg) with a maximal pressure of 30 mmHg in recent studies.

Acellular perfusates were used without the use of an oxygen carrier. Between 1970 and 1986, these were plasma- or albumin-based and often a vasodilator, heparin, antibiotics, corticosteroids, allopurinol, insulin, and buffers to maintain pH were added (Table S6). As of 1980, synthetic perfusates were introduced. Albumin was replaced by synthetic oncotic products (mannitol, hydroxyethyl starch, etc.) and the addition of less permeable anions (like gluconate and lactobionate) prevented further hypothermic-induced cell swelling in contrast with other anions (like chloride) [39]. Adaptations were made to include adenine nucleotide derivates and electrolytes were added in extracellular or intracellular concentrations. After 1986, all studies were performed with synthetic solutions, mostly formulations of Belzer’s Machine Perfusion Solution (MPS) [39]. In some studies, carbohydrates, amino acids, or fatty acids were added to the perfusate (Table S6).

In the majority of studies, the perfusate was oxygenated (40/52; 77%), most commonly by using a membrane oxygenator (26/40; 65%) (Table S2). In these studies, a mixture of O_2_ and CO_2_ [17,19,20,21,22,25,34,40,41,42,43,44,45] or a mixture of O_2_, CO_2_, and nitrogen [26,27,38,46,47,48,49] was given. Surface oxygenation with room air or O_2_ (13/40; 33%) [20,32,37,38,41,42,43,50,51,52,53,54,55] was also used. In one article (3%), bubble and surface oxygenation were combined [20]. Other methods were film oxygenation [43], run-off and tube oxygenation [33,56], hyperbaric oxygenation in a pressure chamber [45], and non-specified oxygenation [28,57]. Some studies investigated different oxygenation types. In the remaining studies, the perfusate was not actively oxygenated (10/52; 19%) [8,15,16,18,30,35,58,59,60,61] or it was not clear if the perfusate was oxygenated (2/52; 4%) [31,62].

### 3.3. Metabolism during Hypothermic Perfusion with Plasma-Based Perfusates

Studies were categorized as either studying carbohydrate (7/9), amino acid (1/9), fatty acid (2/9) metabolism, or the metabolism of high-energy molecules (3/9). All studies used oxygen during perfusion and one study [40] compared oxygenated and non-oxygenated perfusion. Details are listed in Table 1 and Table S7.

#### 3.3.1. Carbohydrate Metabolism

In six studies, minimally injured kidneys were hypothermically perfused with oxygen and all perfusates contained glucose/dextrose at the start [17,23,29,40,44,57]. (Table S6). Studies show increasing [29], decreasing [44], and similar [40] perfusate glucose concentrations over time. The perfusate lactate/pyruvate ratio, said to be an indicator of the redox potential, increased over time [17,23,29,44,57].

When ischemically injured kidneys were perfused with an oxygenated glucose-containing perfusate, glucose concentrations decreased with increasing levels of lactate and pyruvate [25].

#### 3.3.2. Amino Acid Metabolism

Many amino acids (alanine, glutamate, serine, glycine, valine, threonine, (iso)leucine, methionine, aspartate, phenylalanine, lysine, (hydroxy)proline, tyrosine, and arginine) were released during the oxygenated perfusion of minimally injured kidneys [44].

#### 3.3.3. Fatty Acid Metabolism

Perfusate-free fatty acids [29] and lipids [63] decreased during the oxygenated perfusion of minimally injured kidneys. The decrease in perfusate triglycerides and tissue phospholipids was less pronounced when oleate (a long-chain fatty acid) was administered during perfusion, with a more pronounced decrease in perfusate neutral lipids but high retention of tissue triglycerides and neutral lipids [63].

#### 3.3.4. Energy Metabolism

In minimally injured kidneys, ATP levels increased during oxygenated perfusion while in injured kidneys, tissue ATP levels decreased rapidly during warm ischemia, with restoration during oxygenated perfusion [56]. Nevertheless, Pegg et al. did not observe adenine nucleotide restoration [25] and Kahng et al. described a variable range for each adenine nucleotide and a suboptimal energy charge during perfusion [57].

### 3.4. Metabolism during Hypothermic Perfusion with Albumin-Based Perfusates

Studies were categorized as either studying carbohydrate (10/16; 63%), amino acid (2/16; 13%), fatty acid (10/16; 63%) metabolism, or the metabolism of high energy molecules (3/16; 19%). All studies used oxygenated perfusion. Details are listed in Table 2 and Table S8.

In nine studies, radioactively labeled metabolites—also called tracers—were added to the perfusion circuit to investigate active metabolic pathways: ^14^C-glucose [27,42,46,47,48], ^14^C-mevalonate [53], ^14^C-acetate [47], ^14^C-lactate [47], ^14^C-labeled amino acids (^14^C-cycloleucine [42], ^14^C-leucine [41], ^14^C-threonine [41]), ^14^C-labeled fatty acids (^14^C-linoleate [46,48,49], ^14^C-palmitate [26,46,48,49], ^14^C-caprylate [42,46,48], ^14^C-myristic acid [48]).

#### 3.4.1. Carbohydrate Metabolism

A decrease in perfusate glucose and an increase in perfusate lactate concentration was seen during oxygenated perfusion [27,42,46,47,48]. Only one study showed no change in perfusate glucose level with a low lactate and non-measurable pyruvate during oxygenated perfusion with bovine serum albumin [34]. This glucose drop was more pronounced when glucose was a component of the perfusate [27] and less pronounced when amino acids [42] or fatty acids [46,47] were added. Interestingly, Slaattelid et al. also found a different perfusate glucose uptake and release pattern when glucose was administered intravenously before kidney procurement [27]. Three studies found a decrease in tissue glucose regardless of whether the perfusion medium was glucose-rich or -poor [27,37,38]. Tracer studies showed active glucose metabolism, with the detection of ^14^C-CO_2_ and ^14^C-lactate [27,46,47,48]. An increase in the lactate/pyruvate ratio was seen in the studies by Grundmann et al. [23] and Pettersson et al. [48]. Tissue lactate showed no change [38] and was the highest metabolite measured [57], even increasing after 72 h when glucose was added [37].

#### 3.4.2. Amino Acid Metabolism

Many amino acids were released during the oxygenated perfusion of minimally injured kidneys, with an increase in urea and ammonia [42]. The increase was most pronounced for alanine and taurine [41]. No increase was seen for glutamine, proline, aspartate, and cystine [42].

In contrast, when amino acids were added to the perfusate, a decrease in many amino acids (glutamine, proline, glycine, aspartate, arginine, (iso)leucine, methionine, valine, and serine) was seen with an increase only in threonine, tyrosine, ornithine, taurine, and alanine levels [41,42]. Labeled (cyclo)leucine and threonine were incorporated into proteins; more so for leucine which is also reflected in the higher label recovery in CO_2_ from leucine than threonine [41].

#### 3.4.3. Fatty Acid Metabolism

In minimally injured kidneys, a decrease in fatty acids during oxygenated perfusion with a fatty acid-rich perfusate was seen [26,27,38,42,46,47,48,49]. When a defatted perfusate was used, perfusate-free fatty acids increased [24]. Perfusate short-chain fatty acids (caprylate and acetate) decreased rapidly [38,42,46,47,48,49], while a decrease in medium-chain fatty acids (laureate and myristic acid) was seen after a few days when short-chain fatty acids were depleted [46,48]. Labeled short-chain fatty acids were mainly incorporated into CO_2_ [42,46,47,48], glucose [47,48], and lactate [47,48], indicative of fatty acid oxidation and gluconeogenesis. Long-chain fatty acids (palmitate, oleate, linoleate, stearate) decreased more slowly than short- and medium-chain fatty acids [26,38,46,48]. Labeled long-chain fatty acids were incorporated into phospholipids [48,49] and triglycerides [48,49], and to a very low extent into CO_2_ [46,48,49], indicative of utilization as a membrane stabilizer rather than as a source for oxidation.

In tissue, phospholipids decreased with fatty acid-free perfusate [47]. When a fatty acid-rich perfusate was used, phospholipid levels did not change [49] or decreased [48]. Tissue cholesterol levels remained unchanged in three studies in which kidneys were pumped for 2 to 6 days [47,49,53] and decreased in one study after 6 days of perfusion [48].

#### 3.4.4. Metabolism of High-Energy Molecules

In minimally injured kidneys, ATP levels and total adenine nucleotide levels decreased during oxygenated perfusion but the energy charge potential was optimal [37]. While in ischemically injured kidneys, ATP levels increased to near normal after 24 h of oxygenated perfusion but total adenine nucleotide levels remained the same [28] or had a variable range with suboptimal energy charge [57].

### 3.5. Metabolism during Hypothermic Perfusion with Synthetic Perfusates

Studies were categorized as either studying carbohydrate (20/31 65%), amino acid (14/31; 45%), fatty acid (9/31; 29%) metabolism, metabolism of high-energy molecules (23/31; 74%), or TCA cycle metabolism (8/31; 26%). In 19 studies, oxygen was added to the perfusate, in ten studies no oxygen was given, and in two studies it was not clear if oxygen was given.

In two studies, non-radioactively labeled ^13^C-glucose [60,64] was used to further clarify the metabolism. Details are listed in Table 3 and Table S9.

#### 3.5.1. Carbohydrate Metabolism

Perfusion with oxygenated glucose-free perfusate led to low and decreasing levels of tissue glucose and very low and stable lactate levels in minimally injured kidneys [37].

In ischemically injured kidneys, perfused without active oxygenation, glucose changes seem dependent on the glucose concentrations in the perfusate. Indeed, no changes in glucose levels were seen during long-term perfusion with MPS (72 h perfusion with perfusate changes every 24 h) [59]. In a similar 72 h experiment with 24 h renewals of UHK solution, containing less glucose and mannitol compared to MPS, perfusate glucose levels dropped [59]. Similarly, other studies investigating short- and long-term non-oxygenated perfusions of pig kidneys with MPS found no statistical change in perfusate glucose levels [8,16,58] or an increase in glucose [15] with increases in perfusate and tissue lactate [8,15,16]. A similar study with human kidneys showed a glucose and lactate increase in the perfusate [15]. Only one group studied glucose changes during the oxygenated perfusion of injured kidneys and found increased perfusate glucose and lactate levels with similar or increased tissue lactate [19,20,21,22]. Perfusate lactate levels were lower when high oxygen concentrations were given [20,21,22,64].

Tracer studies show active glucose metabolism during (non-)oxygenated perfusion of ischemically injured kidneys. When ^13^C-glucose was infused, ^13^C-lactate appeared in the perfusate and tissue [60,64]. Less labeled lactate was recovered in tissue after highly oxygenated perfusion [64].

Mannitol did not change with non-oxygenated MPS [21] and increased during oxygenated perfusion [21]. Ribose levels remained stable in two studies [8,15] and decreased in one study [16].

#### 3.5.2. Amino Acid Metabolism

Amino acid release in the perfusate was shown for minimally and ischemically injured kidneys, regardless of oxygenation status and a correlation between an increase in amino acids and perfusion duration has been suggested [16]. Glutamate [8,15,16,18,21,58,59,61], alanine [8,15,16,18,21,35,60,61,64], valine [8,15,16,18,35], glycine [8,15,16,18,21], and (iso)leucine [16] were most often studied. A glutamate increase was also seen during hypothermic perfusion with UHK-solution [59]. Glutamate levels were lower in the tissue of highly oxygenated kidneys compared to non- or low-oxygenated kidneys [20,21,22,64].

There is some evidence of metabolization of glucose to amino acids as the infusion of ^13^C-glucose during (non-)oxygenated perfusion resulted in the detection of labeled alanine [60,64] in the perfusate and tissue and labeled glutamate in the tissue [60].

Glutathione (GSH), a tripeptide (cysteine, glycine, glutamate) and powerful antioxidant, is a component of MPS. All studies investigating perfusate GSH showed a decrease during the non-oxygenated and oxygenated perfusion of injured kidneys [8,15,21,35,51,61,64].

Oxidized glutathione in the perfusate also decreased or was undetectable [15,16]. There is some suggestion that higher oxygen concentrations increase the levels of tissue glutathione [64].

#### 3.5.3. Fatty Acid Metabolism

Acetate, a short-chain fatty acid, did not change [8,15,60,64] or increased [35] during non-oxygenated perfusion. Acetate remained unchanged or decreased with oxygenated perfusion [21,64]. The ketone body 3-hydroxybutyrate seems to behave differently in humans (increases) [8,15] and pigs (no change) [8] during non-oxygenated perfusion. All kidneys suffered ischemic injury.

During oxygenated perfusion of minimally injured kidneys with gluconate-based perfusates, tissue phospholipids were studied. An initial decrease was seen in the first 24 h followed by an increase in phospholipids [54].

#### 3.5.4. Energy Metabolism

Belzer’s MPS contains adenine and ribose after studies showed higher ATP concentrations with these additives compared to adenosine during oxygenated perfusion of minimally injured kidneys [32,52,55]. Furthermore, with MPS, ATP content increases with higher oxygen concentrations [50].

In ischemically injured kidneys, ATP content decreased during warm ischemia [30,31,36,65,66] and restoration occurred during perfusion [20,21,22,30,66]. ATP increased more when the perfusate was actively oxygenated [20,21,36,64,65,66]. A similar ATP increase during highly oxygenated perfusion with Celsior [45] and a Haemaccel-based perfusate [37,43] has been shown. An increase in tissue ADP was also observed [21,22]; AMP decreased [21] or remained unchanged [22]. Similar studies in injured kidneys showed an increase in perfusate (hypo)xanthine [8,15,16,21], inosine [8,15,16], and adenosine [16], while no changes [8,15,21] or a decrease [16] in adenine concentration was seen.

#### 3.5.5. TCA Cycle Metabolism

Only a few studies examined TCA cycle intermediates [8,15,16,20,21,22,61,64]. Administration of ^13^C-glucose resulted in the formation of ^13^C-citrate, ^13^C-malate, and ^13^C-succinate in the cortex and medulla, indicative of TCA cycle activity [64]. Tissue ^13^C-succinate was higher if the perfusate was highly oxygenated [64].

## 4. Discussion

Commendable work contributing to our understanding of kidney metabolic behavior during cold perfusion has been performed over the past 50 years. Nevertheless, it remains incompletely understood.

From compiling the findings of this scoping review, and in particular those of “tracer studies” during which a (non)radioactively labeled metabolite is added to the perfusion circuit, it is clear that kidneys are metabolically active during cold perfusion. However, key pieces of the puzzle are missing. The fact that metabolism is intrinsically a complex network of interacting biochemical reactions that can be influenced by numerous factors complicates the interpretation of findings. Indeed, perfusate composition, oxygenation, pre-existing kidney injury, and perhaps even pre-donation nutrient availability seem to influence metabolism during hypothermic perfusion preservation. Furthermore, key enzymes are likely to be influenced by the low temperatures and this in turn will influence metabolism.

Tracer studies have shown the oxidation of glucose, amino acids, and fatty acids to CO_2_ [27,41,42,46,47,48,49] but it is unclear if and how oxygenation levels and pre-existing injury affect this. A few older studies suggest gluconeogenesis from fatty acids [47,48] and the incorporation of amino acids into proteins when these are provided [41]. Indeed, kidney metabolism seems dependent on the perfusate composition. Administration of carbohydrates, amino acids, and lipids seems to change the uptake and release patterns of these metabolites. In almost all studies, glucose was added to the perfusate as an energy source. Interestingly, glucose and lactate metabolism changed when other substrates such as amino acids or fatty acids were added to the perfusate, suggesting these metabolites could have competing interests as energy sources. In vivo, fatty acids and amino acids can feed into the TCA cycle either as acetyl-coenzyme A or another TCA-cycle intermediate and serve as important energy sources [67]. In physiological, in vivo conditions, the kidneys play a role in the synthesis and inter-organ exchange of amino acids [68]. It is unclear how the absence of inter-organ exchange influences amino acid metabolism during cold perfusion. Furthermore, the in vivo renal metabolism of alanine, (iso)leucine, and valine changes during fasting and feeding states [68,69] and this might influence metabolism during cold perfusion as well.

The oxygenation level of the perfusate also seems to affect kidney metabolism during cold perfusion. Indeed, a reasonable number of studies showed differences in kidney metabolism when comparing different oxygen levels. Concerning lactate metabolism, there seems to be a reduced increase in lactate when the perfusate was actively oxygenated compared to non-oxygenated perfusates [21,22,40,43,64]. ATP levels and the supported energy charge are higher when kidneys are oxygenated during perfusion [20,21,36,43,45,50,64,65,66].

Injury levels are likely to play a role as well, but this is less clear as only a few studies directly compared kidneys with different injury levels. Pre-existing warm ischemia depletes ATP levels and studies suggest variable reconstitution of ATP during oxygenated perfusion [28,30,36,43,56,65,66]. Observations of other metabolites in minimally injured kidneys are less clear.

The potential implications of these observations in a clinical setting are important. Indeed, supporting the metabolically active kidney during hypothermic perfusion seems the logical next step. Actively oxygenating the perfusate during the hypothermic perfusion of older DCD kidneys improved graft outcomes compared to standard non-oxygenated hypothermic perfusion in a recent randomized controlled trial [5]. On the other hand, there was no evidence that short (2 h) reconditioning of expanded-criteria donor kidneys, following cold storage, improved graft survival compared to cold storage alone [70]. Perhaps 2 h is too short to change metabolic behavior after a long period of cold storage or perhaps DBD organs respond differently to additional oxygen compared to DCD.

These findings need to be interpreted with a degree of caution. The majority of studies have a considerable risk of bias and studies were conducted over the course of 50 years with numerous changes in perfusate and perfusion conditions. Many studies report on experiments with dog kidneys. It is important to realize that dog kidneys have a higher tolerance to ischemia reperfusion injury, while pig kidneys are more similar to human kidneys [71,72]. Even though pig kidneys are physiologically and anatomically comparable to human kidneys, there is only limited evidence that pig kidney metabolism is the same as that of human kidneys, however, it is reassuring that Nath et al. found comparable metabolites in pig and human kidney perfusates [8].

As with all scoping reviews, it is possible that some relevant articles were not identified or that relevant studies were published after the search. We limited the chance of missing relevant articles by setting up a broad search strategy in collaboration with experienced biomedical reference librarians. Furthermore, the references of included articles were searched to identify any articles that might have been missed in the search.

## 5. Conclusions

In conclusion, kidneys are metabolically active during cold perfusion preservation. This metabolic activity is incompletely understood and is influenced by a multitude of factors including perfusate composition, oxygenation level, and likely pre-existing injury. It is clear that a greater number of well-designed (pre-)clinical studies are necessary to understand this behavior. In the modern era, with increasing DCD donations and the emergence of hypothermic oxygenated perfusion, the focus should be on understanding metabolic perturbations caused by pre-existing injury levels and on the effect of perfusate oxygen levels. The use of tracers in such studies is indispensable to understanding the metabolism, given the complexity of interactions between different metabolites.

## Figures and Tables

**Table 1:** Summary of studies reporting on kidney metabolism with plasma-based perfusate.

Reference	Severity Injury	O_2_	Metabolites Provided in Perfusion Solution at Start	Findings
Carbohydrate metabolism				
Alexander 1970 [17]	Minimal	Yes	acid citrate dextrose, no extra Gluc	P: = in lactate; + in pyruvate; + in lactate/pyruvate ratio (first 30 min) then return to normal
Grundmann 1972 [29]	Minimal	Yes	dextrose	P: + in dextrose (10%); lactate/pyruvate ratio: − (first hours), plateau, + (after 24 h))
Pedersen 1973 [40]	Minimal	Yes/No	Gluc	P: = in Gluc (similar with/without O_2_); − in lactate (greater decrease with O_2_)
Grundmann 1974 [23]	Minimal	Yes	dextrose	P: + in lactate/pyruvate ratio
Kahng 1983 [57]	Injured	Yes	Gluc	T: lactate highest measured metabolite; lactate/pyruvate ratio +/− 70
Pegg 1984 [25]	Minimal/ Injured	Yes	Gluc, mann, caprylate, protein	P: − in Gluc; + in lactate; + in pyruvate
Verkh 1986 [44]	Minimal	Yes	dextrose	P: − in Gluc; + in lactate; + in pyruvate; lactate/pyruvate ratio increased by 11.3%
Amino Acid metabolism				
Verkh 1986 [44]	Minimal	Yes	dextrose	P: + in Ala, Glu, Ser, Gly, Val, Thr, (iso)Leu, Met, Asp, Phe, Lys, (-OH)Pro, Tyr, Arg, His
Fatty Acid metabolism				
Huang 1971 [63]	Minimal	Yes	acid citrate dextrose, mann, sodium oleate	P: no oleate: −25% lipids (TG, less phospholipids); with oleate: − in TG but less vs. no oleate T: no oleate: −35% lipids (neutral lipids and TG − most, total phospholipids − by only 27%); with oleate: phospholipids − by only 8%
Grundmann 1972 [29]	Minimal	Yes	dextrose	P: − in unesterified FA
Energy metabolism				
Collste 1971 [56]	Minimal/ Injured	Yes	acid citrate dextrose, dextrose	T: minimal injury: constant + in ATP level; injury: ATP − during warm ischemia and partial restoration of ATP with perfusion
Kahng 1983 [57]	Injured	Yes	Gluc	T: variable nucleotide contents with wide ranges in each nucleotide
Pegg 1984 [25]	Injured	Yes	Gluc, mann, caprylate, protein	T: no restoration of adenine nucleotides

AA, amino acids; FA, fatty acids; Gluc, glucose; mann, mannitol; P, perfusate; T, tissue; TG, triglyceride; O_2_, oxygen; +, increase; −, decrease; =, no change. Minimally injured: <5 min warm ischemia and <30 min cold ischemia; injured: all other cases.

**Table 2:** Summary of studies reporting on kidney metabolism with an albumin-based perfusate.

Reference	Severity Injury	O_2_	Metabolites Provided in Perfusion Solution at Start	Findings
Carbohydrate metabolism				
Pegg 1972 [34]	Minimal	Yes	Gluc	P: = in Gluc; no measurable + pyruvate; lactate levels remained below 0.5 mM
Grundmann 1974 [23]	Minimal	Yes	FA-rich (human albumin), (labeled) Gluc, acetate, lactate	P: + in lactate/pyruvate ratio; perfusate exchange
Pettersson 1974 [48]	Minimal	Yes	Gluc-rich (Gluc), octanoate	P: − in Gluc; + in lactate and lactate/pyruvate ratio Label (P): Gluc label mainly seen in lactate and to a low extent in glycogen and CO_2_
Lundstam 1975 [46]	NA	Yes	(labeled) Gluc, linoleate, palmitate, myristic acid, caprylate	P: − in Gluc; + in lactate (lower in FA-free with higher Gluc oxidation) Label (P): modest incorporation of Gluc label in CO_2_, similar findings human and dog
Slaattelid 1976(2) * [27]	Minimal	Yes	Gluc-free (mann)	P: + in Gluc if animal received Gluc infusion before procurement; T: − in Gluc, − in renal glycogen, higher glucose when Gluc infusion before procurement
Slaattelid 1976(2) * [27]	Minimal	Yes	(labeled) Gluc, linoleate, palmitate, caprylate	P: − in Gluc; + in lactate; Label (P): − in labeled Gluc with modest (3%) metabolization to lactate and CO_2;_ T: high concentration of Gluc
Lundstam 1976 * [47]	Minimal	Yes	FA-free (bovine albumin), (labeled) Gluc, lactate	P: − in Gluc (mainly initially) + in lactate (lowest in FA free perfusate) Label (P): incorporation of Gluc carbon into CO_2_ (greater in FA-free perfusate the first 3 days); − in labeled lactate with recovery in CO_2_ and Gluc
Lundstam 1976 * [47]	Minimal	Yes	FA-rich (human albumin), (labeled) Gluc, acetate and lactate	P: constant − in Gluc; + in lactate (most pronounced with acetate, FA-rich perfusate) Label (P): incorporation of Gluc carbon into CO_2_ (less in FA-rich perfusate) − in labeled lactate with recovery in CO_2_ and Gluc
Lundstam 1977(2) * [42]	Minimal	Yes	AA (labeled Gluc)	P: slight − in Gluc; + in lactate (first 4 days), plateau or decrease hereafter; Label (P): − in specific activity Gluc
Lundstam 1977(2) * [42]	Minimal	Yes	no AA, (labeled) Gluc, caprylate, cycloleucine	P: greater − in Gluc; continuous + in lactate
Fischer 1979 [38]	Minimal	Yes	dextrose	T: − in Gluc (non-significant); no + in lactate
Fischer 1980 [37]	Minimal	Yes	Gluc, caprylate	T: significant − in Gluc; stable lactate levels up to 72 h with + thereafter
Kahng 1983 [57]	Injured	Yes	Gluc	T: lactate highest measured metabolite; lactate/pyruvate ratio +/− 70
Amino Acid metabolism				
Lundstam 1977(1) [41]	Minimal	Yes	Gluc, 17 L-AA *, (labeled) leucine, threonine	P: − in Leu, slight + in Thr; Label (P): − in specific activity Leu and Thr; Label (T): carbon incorporation Leu > Thr in protein, Carbon Leu >> Thr incorporation in CO_2_
Lundstam 1977(2) * [42]	Minimal	Yes	17 L-AA *,(labeled) Gluc, caprylate, cycloleucine	P: rapid − in Gln, Pro, Gly, Asp, Arg, slower − in (iso)Leu, Met, Val; − in Ser; = His, Cys, Lys, Phe; + for Thr, Tyr, Orn, Ala, taurine, ammonia and urea, + in Glu first days, − after 4th day
Lundstam 1977(2) * [42]	Minimal	Yes	no AA, (labeled) Gluc, caprylate, cycloleucine	P: + in almost all AA and ammonia (most pronounced for Ala and taurine) no + for Gln, Pro, Asp, Cys; + in nitrogen
Fatty acid metabolism				
Pettersson 1974 [48]	Minimal	Yes	(labeled) Gluc, linoleate, palmitate, myristic acid, caprylate	P: − in FFA (fast − in short-chain FA (caprylate, after 2 days − in middle-chain FA (laureate and myristic acid), slow − in long-chain FA (palmitate, oleate, linoleate, stearate); Label (P): high incorporation of caprylate label in Gluc and lactate; T: = in TG and phospholipids; − cholesterol after 6 days; Label (T): caprylate mostly oxidized to CO_2_ until day 4, then + oxidation of myristic acid to CO_2_; low incorporation of labeled palmitate into CO_2_; labeled linoleate, palmitate and myristic acid incorporated mainly in phospholipids and TG
Lundstam 1975 [46]	NA	Yes	(labeled) Gluc, linoleate, palmitate, caprylate	P: fast − in FA (fast − caprylate, slow followed by fast − (after 2–4 days) for laureate and myristic acid and slow − in palmitate, oleate and linoleate); Label (P): fast incorporation of caprylate in CO_2_ (first 3 days), from day 4 faster incorporation of myristic acid label in CO_2_, low incorporation of label from long-chain FA in CO_2_
Halasz 1975 [24]	Minimal	Yes	mann, defatted albumin	P: + in FFA
Lundstam 1976 * [47]	Minimal	Yes	FA-free (bovine albumin), (labeled) Gluc, lactate	T: − in phospholipids
Lundstam 1976 * [47]	Minimal	Yes	FA-rich (human albumin), (labeled) Gluc, acetate and lactate	Label (P): − in labeled acetate with incorporation in CO_2_ (first 2–4 days), Gluc and lactate; T: = in cholesterol
Slaattelid 1976(1) * [26]	Minimal	Yes	FFA-rich, (labeled) palmitate, Gluc	P: fast linear − in FFA; Label (P): slow − in labeled palmitate
Slaattelid 1976(1) * [26]	Minimal	Yes	FFA-poor, (labeled) Gluc	P: low FFA was remained
Slaattelid 1976(2) [27]	Minimal	Yes	Gluc-free (mann)/Gluc-rich (labeled Gluc)	P: − in FFA
Lundstam 1977(2) [42]	Minimal	Yes	17 L-AA **/no AA,(labeled) Gluc, caprylate, cycloleucine	P: higher − in FA with AA; rapid − in caprylate (depleted day 3–4); Label (P): high incorporation of labeled caprylate in CO_2_
Fischer 1979 [38]	Minimal	Yes	Gluc, caprylate	P: first 24 h: − in caprylate; no − in long-chain FFA (+ in palmitate and oleate, no − in stearate and linoleate); after 24 h: depletion of caprylate and − in long-chain FFA
Skrede 1979 [49]	Minimal	Yes	Gluc, labeled palmitate and linoleate, caprylate	P: − in caprylate, + in all long-chain FA and arachidonic acid; Label (P): only traces of palmitate and linoleate label in CO_2_; T: 10% − in total phospholipids, − in all FA except arachidonic acid, = in cholesterol and TG; Label (T): palmitate and linoleate label in tissue lipids (higher in phospholipids than TG)
Kleist 1982 [53]	Minimal	Yes	Gluc, 17 L-AA **	Label (P): − in mevalonate label, only small amounts in CO_2;_ T: no − in cholesterol when mevalonate added; Label (T): mevalonate label in total lipid fraction of kidney cortex, (80% recovered in cholesterol and cholesterol precursors and 20% in FA containing lipids)
Energy metabolism				
Collins 1977 * [28]	Minimal	Yes	mann	T: = in TAN levels during 72 h perfusion
Collins 1977 * [28]	Injured	Yes	mann	T: warm ischemia: − in ATP, ADP and TAN (first 15′ + in AMP, thereafter −); perfusion: regeneration of ATP and rise in energy charge (ATP+ 1/2 ADP/TAN) to near normal within first hour (no significant regeneration of TAN)
Fischer 1980 [37]	Minimal	Yes	Gluc-rich (Gluc), octanoate	T: − in TAN and ATP; energy charge was optimal in both groups, significantly lower without Gluc after 72 h)
Kahng 1983 [57]	Injured	Yes	Gluc	T: variable nucleotide contents with wide ranges in each nucleotide

AA, amino acids; (F)FA, (free) fatty acids; Gluc, glucose; mann, mannitol; P, perfusate; T, tissue; TG, triglyceride; TAN, total adenine nucleotides (ATP + ADP + AMP). +, increase; −, decrease; =, no change. *, this paper reports on experimental groups with different perfusates and these are presented separately in this table. 17 L-AA **: aspartate, alanine, arginine, glycine, histidine, isoleucine, leucine, lysine, methionine, phenylalanine, proline, serine, threonine, tryptophan, valine, glutamine, tyrosine. Minimally injured: <5 min warm ischemia and <30 min cold ischemia; injured: all other cases.

**Table 3:** Summary of studies reporting on kidney metabolism with a synthetic perfusate.

Reference	Severity Injury	O_2_	Metabolites Provided in Perfusion Solution at Start	Findings
Carbohydrate metabolism				
Fischer 1980 [37]	Minimal	Yes	Gluc-free (mann)	T: low Gluc and slight − in Gluc; stable lactate levels (+/− zero) without +
Pegg 1981 [43]	Minimal/ Injured	Yes	Haemaccel-based; Gluc (+/− caprylate, acetate, pyruvate)	P: − Gluc utilization with acetate/pyruvate, − Gluc with caprylate, less lactate accumulation with higher pO_2_ and/or hypoxanthine, + in lactate and pyruvate with caprylate
Pegg 1984 [25]	Injured	Yes	Haemaccel-based; Gluc, mann, caprylate, protein	P: − in Gluc; + in lactate; + in pyruvate
Baicu 2004 [58]	Injured	No	MPS (Gluc, mann, rib, GSH, adenine), +/− fructose-1,6-biphosphate (FDP)	P: = in Gluc; = in pyruvate (constant low value) Interstitial fluid (micro dialysis): + in pyruvate (higher concentrations FDP- kidneys)
Baicu 2006 * [59]	Injured	No	MPS (Gluc, mann, rib, GSH, adenine)	P: = in Gluc, perfusate replacement every 24 h
Baicu 2006 * [59]	Injured	No	Unisol-UHK, sucrose, mann, Gluc, GSH, adenosine	P: − in Gluc (perfusate replacement every 24 h)
Bon 2014 [18]	Injured	No	MPS (Gluc, mann, rib, GSH, adenine)	P: + in lactate
Nath 2014 [8]	Injured	No	MPS (Gluc, mann, rib, GSH, adenine)	P: = in Gluc; + in lactate; = in mann and rib; pig and human comparable
Guy 2015 [15]	Injured	No	MPS (Gluc, mann, rib, GSH, adenine)	P: + in Gluc; + in lactate; = in mann and rib
Nath 2016(1) [60]	Injured	No	MPS ((labeled)Gluc, mann, rib, GSH, adenine)	P: + in lactate; Label (P): + in labeled lactate from labeled Gluc; Label (T): labeled lactate present in kidney cortex
Nath 2016(2) [61]	Injured	No	MPS (Gluc, mann, rib, GSH, adenine)	P: + in lactate; T: lactate in HMP was lower than at start; Total metabolite amount (T + P): + in lactate
Hamaoui 2016 [62]	Injured	NA	Belzer-UWMP, raffinose, GSH, adenosine	P: + in Gluc levels (first 3 h), − thereafter; + in lactate Micro dialysis: + in cortical lactate (after 1.5 h)
Ravaioli 2018 [45]	Injured	No/Yes	Celsior, mann, glutamate, histidine, GSH	P: + in lactate
Darius 2018 [19]	Injured	No/Yes	MPS (Gluc, mann, rib, GSH, adenine)	P: + in Gluc (no difference O_2_ vs. non- O_2_)
Patel 2019 [64]	Injured	Yes	MPS (Gluc, mann, rib, GSH, adenine) + (labeled)Gluc	Label (P): + in labeled lactate from labeled Gluc (less + in O_2_ vs. steady + in air); Label (T): lower cortical concentrations of labeled lactate in O_2_ vs. air.
Darius 2020(1) [21]	Injured	Yes/No	MPS (Gluc, mann, rib, GSH, adenine)	P: + in lactate and + in mann (in groups with pre-or end-O_2_) (all O_2_ strategies resulted in lower concentrations of lactate) no differences in perfusate Gluc between O_2_ conditions; T: = in lactate
Darius 2020(2) [22]	Injured	Yes/No	MPS (Gluc, mann, rib, GSH, adenine)	P: + in Gluc (independent of O_2_ content); lower lactate in HMPO_2_ high vs. HMP; T: + in lactate
Darius 2020(3) [20]	Injured	Yes/No	MPS (Gluc, mann, rib, GSH, adenine)	P: + in Gluc (independent of O_2_ content); T: = in lactate
Faucher 2022 [16]	Injured	No	MPS (Gluc, mann, rib, GSH, adenine)	P: = in Gluc and mann; + in lactate; − in rib
Mrakic-Sposta 2023 [35]	Injured	No	MPS (Gluc, mann, rib, GSH, adenine)	P: + in lactate; T: + in lactate
Amino Acid metabolism				
Boudjema 1991 [51]	Minimal	Yes	UWMP(Gluc, GSH, raffinose, rib, adenine, adenosine)	P: reduced GSH disappeared from perfusate in 24 h; T: loss of GSH from the cortex tissue, if reduced GSH administered then less GSH loss in tissue; adding glycine, glutamate, cysteine stimulated GSH synthesis while GSSG did not prevent GSH loss.
Baicu 2004 [58]	Injured	No	MPS (Gluc, mann, rib, GSH, adenine), +/− fructose-1,6-biphosphate (FDP)	P: + in Glu
Baicu 2006 * [59]	Injured	No	MPS (Gluc, mann, rib, GSH, adenine)	P: + in Glu and ammonia (the first hours)
Baicu 2006 * [59]	Injured	No	Unisol-UHK, sucrose, mann, Gluc, GSH, adenosine	P: Baseline Gln detected; + in Glu (higher in UHK than Belzer MPS) + in NH4+ (higher in UHK than Belzer MPS)
Bon 2014 [18]	Injured	No	MPS (Gluc, mann, rib, GSH, adenine)	P: + in Val, Ala, Gly, Glu
Nath 2014 [8]	Injured	No	MPS (Gluc, mann, rib, GSH, adenine)	P: + in Gly, Glu, Ala, (iso)Leu, Val; = in Tyr; − in GSH
Guy 2015 [15]	Injured	No	MPS (Gluc, mann, rib, GSH, adenine)	P: + in Ala, Gly, Glu, (iso)Leu, Tyr, Val; − in reduced GSH; GSSG not detected
Nath 2016(1) [60]	Injured	No	MPS ((labeled)Gluc, mann, rib, GSH, adenine)	P: + in Ala; Label (P): + in labeled Ala from labeled Gluc; Label (T): labeled Ala from Gluc present in kidney cortex, labeled Glu in small amounts (<0.5% of total Glu)
Nath 2016(2) [61]	Injured	No	MPS (Gluc, mann, rib, GSH, adenine)	P: + in Ala, Glu; − in reduced GSH; T: Absence of reduced GSH Total metabolite amount (T + P): + in Glu, Ala, Asp, Leu, Tyr
Patel 2019 [64]	Injured	Yes	MPS (Gluc, mann, rib, GSH, adenine) + (labeled)Gluc	P: Ala (+ air/= O_2_), Asp (= air/+ O_2_), Glu (= air/− O_2_), Glyl (= air/O_2_); − in GSH (in both oxygenation conditions); Label (P): + in labeled Ala (reduction in + in O_2_ vs. steady + in air); T: higher concentrations of Asp, Tyr, Val, Gly; Ala in cortex in O_2_ vs. air, lower Gllu in cortex in O_2_ vs. air; higher cortex GSH in O_2_ vs. air; Label (T): higher concentrations of [4,5-^13^C] Glu in cortex in O_2_ vs. air
Darius 2020(1) [21]	Injured	Yes/No	MPS (Gluc, mann, rib, GSH, adenine)	P: + in Ala, Asp, Gly, (iso)Leu, Glu; − in GSH (pre/end O_2_); T: − in Glu (lower in O_2_)
Darius 2020(2) [22]	Injured	Yes/No	MPS (Gluc, mann, rib, GSH, adenine)	P: GSH (no difference between O_2_ conditions) T: = in Glu (lower in O_2_)
Darius 2020(3) [20]	Injured	Yes/No	MPS (Gluc, mann, rib, GSH, adenine)	T: − in Glu (lower when O_2_ was added for 2 h by membrane oxygenator than with bubble and intermittent surface oxygenation or when no O_2_ was given)
Faucher 2022 [16]	Injured	No	MPS (Gluc, mann, rib, GSH, adenine)	P: + in Trp, Asp, Ser, Gly, Thr, Glu, Ala, (Orn), Pro, Lys, His, Arg, Val, Met, Tyr, (iso)Leu, Phe, taurine; + in AA correlated with perfusion duration; = in GSH (reduced form from MPS); − in GSSG
Mrakic-Sposta 2023 [35]	Injured	No	MPS (Gluc, mann, rib, GSH, adenine)	P: + in Val, Ala; − in total GSH levels; T: + in Val, Ala
Fatty acid metabolism				
Southard 1984(1) [54]	Minimal	Yes	Gluconate based; Gluc, GSH	T: − in phospholipids (first 24 h) thereafter +; isolated mitochondria: initial − in phospholipids with + after 3 days (FFA show gradual +)
Nath 2014 [8]	Injured	No	MPS (Gluc, mann, rib, GSH, adenine)	P: = in acetate (human + pig); + (human), = (pig) in 3-hydroxybutyrate
Guy 2015 [15]	Injured	No	MPS (Gluc, mann, rib, GSH, adenine)	P: = in acetate; + in the ketone body 3-hydroxybutyrate
Nath 2016(1) [60]	Injured	No	MPS ((labeled)Gluc, mann, rib, GSH, adenine)	P: = in acetate; Label (P): = in labeled acetate from labeled Gluc (concentration = 1.25% at all time points)
Nath 2016(2) [61]	Injured	No	MPS (Gluc, mann, rib, GSH, adenine)	Total metabolite amount (T + P): + in acetate
Patel 2019 [64]	Injured	Yes	MPS (Gluc, mann, rib, GSH, adenine) + (labeled)Gluc	P: − air = O_2_ in acetate; T: higher cortical concentration of acetate in O_2_
Darius 2020(1) [21]	Injured	Yes	MPS (Gluc, mann, rib, GSH, adenine)	P: − in acetate (pre-or end-O_2_)
Darius 2020(2) [22]	Injured	Yes	MPS (Gluc, mann, rib, GSH, adenine)	P: acetate (lower in O_2_ groups)
Mrakic-Sposta 2023 [35]	Injured	No	MPS (Gluc, mann, rib, GSH, adenine)	P: + in acetate
Energy metabolism				
Fischer 1980 [37]	Minimal	Yes	Gluc-free (mann)	T: − in TAN and ATP; energy charge (EC= ATP +1/2 ADP/TAN) optimal in both groups, (EC lower in the group without Gluc after 72 h)
Pegg 1981 [43]	Minimal/ Injured	Yes	Haemaccel-based; Gluc (+/− caprylate, acetate, pyruvate)	T: − in TAN, ATP, ATP/ADP ratio with WI (high-energy phosphate stores depleted when Gluc was sole energy source and pO_2_ 150 mmHg) When O_2_ tension was 600 mmHg and with Gluc, caprylate, hypoxanthine added, 5′nucleotide adenine levels maintained close to normal values; with 60′ WIT: total AN level was restored to normal (only the ATP/ADP ratio was depressed) after 48 h of perfusion
Pegg 1984 [25]	Injured	Yes	Haemaccel-based; Gluc, mann, caprylate, protein	T: significant AN restoration
Southard 1984(2) [55]	Minimal	Yes	Gluconate based; Gluc, GSH, no/adenosine	P: − in adenosine; T: − in ATP (loss can be prevented by including both adenosine (10 mM) and PO4 (25 mM)
Southard 1984(3) [52]	Minimal	Yes	Gluconate based, Gluc, GSH, adenosine	T: higher ATP content in cortex tissue after 3 days perfusion than control (perfusion with adenosine and PO4), concentration of ATP in cortex tissue from 5-day perfused kidneys was less than control
McAnulty 1988 [32]	Minimal	Yes	Gluconate based, Gluc, GSH, rib + adenine/adenosine	P: almost complete degradation of adenosine (5 d); + in hypoxanthine and inosine; only 10% loss of adenine (no large + in purine end products); T: higher ATP and TAN in cortical tissue in adenine/rib kidneys than in adenosine kidneys after 5 days
Minor 2005 * [33]	Injured	Yes	MPS (Gluc, mann, rib, GSH, adenine)	T: improved energy status with O_2_ perfusion with Belzer (2.43 +- 0.23 µmol ATP/g) vs. HTK (1.18 +- 0.12 µmol ATP/g)
Minor 2005 * [33]	Injured	Yes	HTK (mann, histidine, tryptophan	T: improved energy status with O_2_ perfusion with Belzer (2.43 +- 0.23 µmol ATP/g) vs. HTK (1.18 +- 0.12 µmol ATP/g)
La Manna 2009 [31]	Injured	NA	Belzer solution, not further specified	T: − in ATP levels
Buchs 2011 [65]	Minimal/ Injured	Yes	MPS (Gluc, mann, rib, GSH, adenine)	T: + in ATP (only with O_2_ perfusion); − in ATP with WI
Lazeyras 2012 [50]	Minimal	Yes	MPS (Gluc, mann, rib, GSH, adenine)	T: ATP depletion during cold storage with almost complete ATP recovery during cold perfusion; ATP detection only with higher O_2_ concentrations
Nath 2014 [8]	Injured	No	MPS (Gluc, mann, rib, GSH, adenine)	P: + in hypoxanthine and inosine; = in adenine
Guy 2015 [15]	Injured	No	MPS (Gluc, mann, rib, GSH, adenine)	P: + in inosine and hypoxanthine; = in adenine
Nath 2016(2) [61]	Injured	No	MPS (Gluc, mann, rib, GSH, adenine)	Total metabolite amount (T + P): + in hypoxanthine; − in inosine
Ravaioli 2018 [45]	Injured	No/Yes	Celsior, mann, glutamate, histidine, GSH, ketoglutarate)	T: − in ATP (non-O_2_ perfusion); + in ATP (O_2_ and hyperbaric perfusion); ATP higher in O_2_ and hyperbaric vs. non-O_2_ perfusion
Patel 2019 [64]	Injured	Yes	MPS (Gluc, mann, rib, GSH, adenine) + (labeled)Gluc	T: higher ATP, ADP in cortex in O_2_ vs. air; AMP comparable between O_2_ vs. air; no difference in adenosine between O_2_ and air
Kaminski 2019 [30]	Injured	No	MPS (Gluc, mann, rib, GSH, adenine)	T: decrease in ATP with 60′ WI; + in ATP with hypothermic perfusion after 60′ WI
Venema 2019 [36]	Injured	No/Yes	MPS (Gluc, mann, rib, GSH, adenine)	T: ATP depletion after 30′ WI; no + in ATP during 24 h non-O_2_ perfusion; + in ATP during 24 h O_2_ (21% or 100%) perfusion
Darius 2020(1) [21]	Injured	Yes	MPS (Gluc, mann, rib, GSH, adenine)	P: + in hypoxanthine; = in adenine (pre- or end O_2_); T: + in ATP and ADP in pre-O_2_ group; − in AMP in all groups; no differences in AMP in O_2_ vs. non-O_2_ group
Darius 2020(2) [22]	Injured	Yes	MPS (Gluc, mann, rib, GSH, adenine)	P: no difference in adenine, hypoxanthine with different O_2_ conc; T: + in ATP and ADP; = in AMP; no differences in ATP, ADP and AMP with different O_2_ conc
Darius 2020(3) [20]	Injured	Yes	MPS (Gluc, mann, rib, GSH, adenine)	T: + in ATP and ADP in O_2_ groups; − in AMP; ATP higher in O_2_ vs. no O_2_; no difference in ADP, AMP in O_2_ vs. non- O_2_
Longchamp 2020 [66]	Min/Inj	Yes	MPS (Gluc, mann, rib, GSH, adenine)	T: ATP + (in the presence of O_2_); 0′ WI: ATP remained stable up to 22 h of perfusion; − in PME; 60′ WI: − in total ATP but = in PME (containing AMP)
Faucher 2022 [16]	Injured	No	MPS (Gluc, mann, rib, GSH, adenine)	P: + in inosine, xanthosine, (hypo)xanthine and adenosine; − in adenine and rib
TCA cycle metabolites				
Nath 2014 [8]	Injured	No	MPS (Gluc, mann, rib, GSH, adenine)	P: + in fumarate; = in citrate
Guy 2015 [15]	Injured	No	MPS (Gluc, mann, rib, GSH, adenine)	P: + in citrate
Nath 2016(2) [61]	Injured	No	MPS (Gluc, mann, rib, GSH, adenine)	Total metabolite amount (T + P): + in fumarate, succinate
Patel 2019 [64]	Injured	Yes	MPS (Gluc, mann, rib, GSH, adenine) + (labeled)Gluc	P: no difference in fumarate between O_2_ conditions; T: no difference in fumarate between O_2_ conditions; Label (T): Higher labeled succinate in O_2_ vs. air, labeling of citrate and malate in cortex and medulla
Darius 2020(1) [21]	Injured	Yes	MPS (Gluc, mann, rib, GSH, adenine)	P: = in succinate (pre- or end O_2_); T: − in succinate (in O_2_ group)
Darius 2020(2) [22]	Injured	Yes	MPS (Gluc, mann, rib, GSH, adenine)	P: succinate (lower in O_2_); T: − in succinate (lower in O_2_ groups)
Darius 2020(3) [20]	Injured	Yes	MPS (Gluc, mann, rib, GSH, adenine)	T: − in succinate (lower in 2 h O_2_ group)
Faucher 2022 [16]	Injured	No	MPS (Gluc, mann, rib, GSH, adenine)	P: + in alpha-keto-glutarate

AA, amino acids; (F)FA, (free) fatty acids; Gluc, glucose; mann, mannitol; rib, ribose; conc, concentration; P, perfusate; T, tissue; TG, triglyceride; TAN, Total Adenine Nucleotide (ATP + ADP + AMP); GSH, glutathione; GSSG, oxidized glutathione; WI, warm ischemia; FDP, fructose-1,6 diphosphate; MPS, Machine perfusion solution (Belzer); HTK, perfusion solution (see Table S6); UHK, perfusion solution (see Table S6); EC, energy charge; AN, adenine nucleotide; PME, phosphomonoester (contains AMP peak). +, increase; −, decrease; =, no change. *, this paper reports on experimental groups with different perfusates and these are presented separately in this table. Minimally injured: <5 min warm ischemia and <30 min cold ischemia; injured: all other cases.

## Data Availability

The dataset has been deposited in RDR, KU Leuven’s data repository, and is publicly available via https://doi.org/10.48804/AMSYVO [12].

## Supplementary Materials

The following supporting information can be downloaded at: https://www.mdpi.com/article/10.3390/jcm12113613/s1, Figure S1 Flow chart of the systematic search identifying published papers reporting on kidney metabolism during hypothermic perfusion; Figure S2: Risk of bias assessment in 30 studies identified, using SYRCLEs tool [13]; Table S1: Search string in databases Pubmed, Embase, Cochrane Library and Web of Science Core Collection; Table S2: Inclusion and exclusion criteria; Table S3: Overview of study set-up and perfusion characteristics; Table S4: Detailed Risk of Bias Assessment using SYRCLE’s tool for articles reporting on animal studies; Table S5: Quality assessment of studies including human kidneys according to the NIH quality assessment score; Table S6: Composition of different perfusion solutions studied in this review; Table S7: Extended summary of studies reporting on kidney metabolism with plasma-based perfusates; Table S8: Extended summary of studies reporting on kidney metabolism with albumin-based perfusates; Table S9: Extended summary of studies reporting on kidney metabolism with synthetic perfusates. Refs. [73,74] are cited in the Supplemental File.
