# Supplementary material for: Current Insights into the Metabolome during Hypothermic Kidney Perfusion—A Scoping Review
Source: J Clin Med. 2023 May 23;12(11):3613. doi: 10.3390/jcm12113613 (PMC10253488; doi:10.3390/jcm12113613)
Supplement: Supplementary file 1 [file jcm-12-03613-s001.zip › jcm-2380396-supplementary.pdf]

Supplementary materials to

Current Insights into the Metabolome during Hypothermic Kidney Perfusion—A Scoping Review

Authors: Laurence Verstraeten [1], Rutger Den abt [1], Bart Ghesquière [2,3], Ina Jochmans [1,4,*]

Affiliations:

1 Laboratory of Abdominal Transplantation KU Leuven, Transplantation Research Group, Department of Microbiology, Immunology and Transplantation, KU Leuven, 3000 Leuven, Belgium
2 Metabolomics Expertise Center, Center for Cancer Biology, VIB, 3000 Leuven, Belgium
3 Laboratory of Applied Mass Spectrometry, Department of Cellular and Molecular Medicine, KU Leuven, 3000 Leuven, Belgium
4 Department of Abdominal Transplant Surgery, University Hospitals Leuven, 3000 Leuven, Belgium
* Correspondence: ina.jochmans@kuleuven.be

Content

Figure S1: Study Flow chart…… P2
Figure S2: Risk of bias assessment…… P2
Table S1: Search string in databases Pubmed, Embase, and Web of Science Core Collection……. P3
Table S2: Inclusion and exclusion criteria…… P4
Table S3: Overview of study set-up and perfusion characteristics…… P5
Table S4: Detailed Risk of Bias Assessment using SYRCLE's tool for articles reporting on animal studies…… P9
Table S5: Quality assessment of studies including human kidneys according to the NIH quality assessment score…… P21
Table S6: Composition of different perfusion solutions studied in this review…… P22
Table S7: Extended summary of studies reporting on kidney metabolism with plasma based perfusate…… P26
Table S8: Extended summary of studies reporting on kidney metabolism with albumin based perfusate…… P27
Table S9: Extended summary of studies reporting on kidney metabolism with synthetic perfusate…… P30
References…… P36

Supplementary Figures

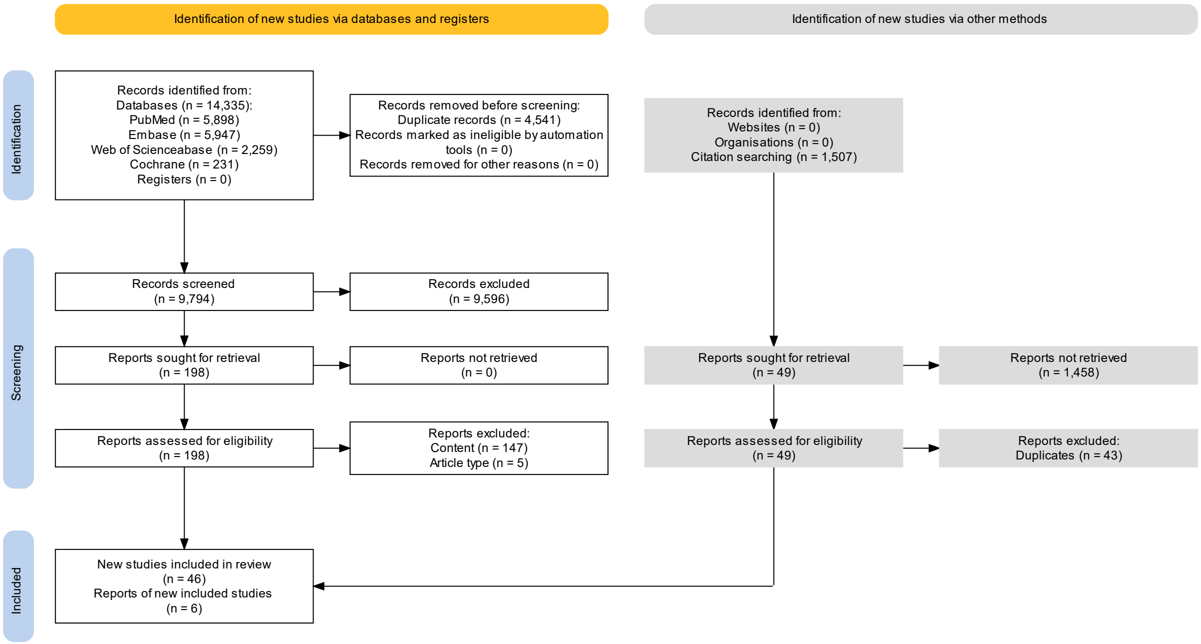

Figure S1: Study Flow chart

A systematic search of online databases, performed on February 2nd, 2023, resulted in the identification of 14,335 articles. After duplicate removal, 9794 articles remained of which 9596 articles were excluded based upon predefined in and exclusion criteria at time of initial record screening (title and abstract screening): 479 based on language, 51 based on type of publication, and 9051 based on content. Another 152 articles were excluded at time of full text screening, leaving 46 articles that were included. From the reference lists, another 1507 potential papers were identified leading to 6 additional inclusions. In total, 52 papers were included in this scoping review.

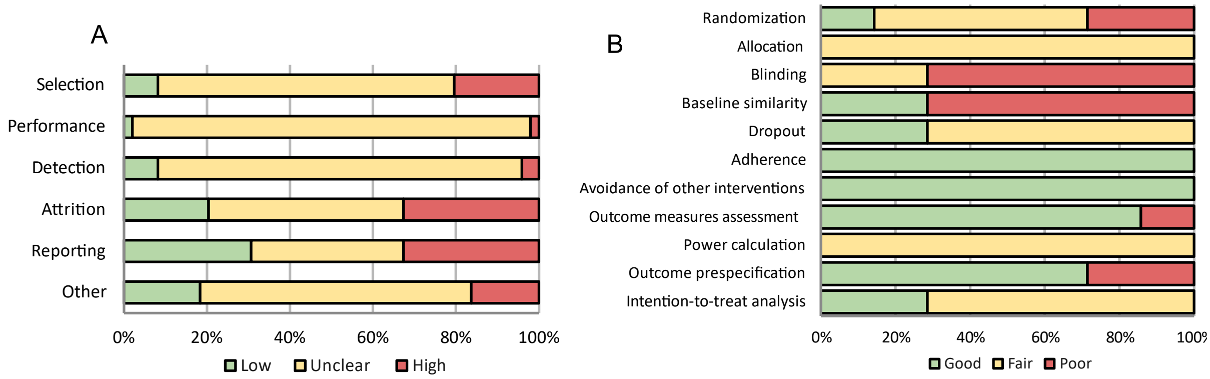

Figure S2: Risk of bias assessment

(A) Risk of bias assessment making use of SYRCLEs tool for animal studies (low, unclear, or high risk) [1]; (B) Risk of bias assessment making use of the NIH tools for human studies (good, fair, or poor quality) [2].

Supplementary Tables

Table S1: Search string in databases Pubmed, Embase, Cochrane Library, and Web of Science Core Collection

Database	Search string
**PubMed**	("Metabolomics"[Mesh] OR "Metabolism"[Mesh] OR "metabonom*"[tiab] OR "metabol*"[tiab] OR "anabol*"[tiab] OR "catabol*"[tiab] OR "lipidom*"[tiab] OR "proteom*"[tiab]) AND ("Kidney"[Mesh] OR "Kidney Transplantation"[Mesh] OR "kidney*"[tiab] OR "renal"[tiab]) AND ("Perfusion"[Mesh:noexpl] OR "perfus*"[tiab]).
**Embase**	(’Metabolomics’/de OR ’Metabonomics’/de OR ’Metabolism’/exp OR ’metabonom*’:ti,ab,kw OR ’metabol*’:ti,ab,kw OR ’anabol*’:ti,ab,kw OR ’catabol*’:ti,ab,kw OR ’lipidom*’:ti,ab,kw OR ’proteom*’:ti,ab,kw) AND (’Kidney perfusion’/exp OR ’Kidney transplantation’/exp OR ’kidney*’:ti,ab,kw OR ’renal’:ti,ab,kw) AND (‘Kidney perfusion’/exp OR ‘perfus*’:ti,ab,kw) AND ('article'/it OR 'article in press'/it OR 'conference paper'/it OR 'conference review'/it OR 'preprint'/it OR 'review'/it) NOT ('chapter'/it OR 'conference abstract'/it OR 'editorial'/it OR 'erratum'/it OR 'letter'/it OR 'note'/it OR 'short survey'/it OR 'tombstone'/it).
**Cochrane Library**	([mh "Metabolism"] OR [mh "Metabolomics"] OR (Metabonom* OR metabol* OR anabol* OR catabol* OR lipidome* OR proteome*)ti,ab,kw) AND ([mh "Kidney"] OR [mh "Kidney Transplantation"] OR (Kidney* OR renal)ti,ab,kw) AND ([mh "Perfusion"] OR (Perfus*)ti,ab,kw).
**Web of Science**	TS=(”metabonom*” OR ”metabol*” OR ”anabol*" OR ”catabol*" OR ”lipidom*" OR ”proteom*") AND TS=("kidney*" OR "renal") AND TS=(“perfus*”).

Table S2: Inclusion and exclusion criteria

**Inclusion criteria**	1. Language: research articles in English, Dutch or French<br>2. Research articles: original research articles, systematic reviews without any restrictions of publication date or specific journals<br>3. Content:<br>a) Study must be performed in following species (mammals: rodents, dogs, pigs, humans)<br>b) Only studies examining kidneys will be included<br>c) Kidneys have to undergo hypothermic (≤12°C) [3] perfusion while being isolated from the rest of the body (i.e. either ex situ or in situ but with clear description of surgical isolation from the rest of the body/vasculature)<br>d) The metabolites studied should be endogenous<br>e) Study outcomes should be based on unravelling/evaluating kidney metabolism<br>4. Full text available (freely online or via KU Leuven Association)
**Exclusion criteria**	1. Language: other than English, Dutch, French<br>2. Study type: all papers that are not original research articles or systematic review, e.g., review articles, letter to editor, conference abstracts, editorials, …<br>3. Content:<br>a) Studies not using mammal kidneys (e.g. cell cultures)<br>b) Only organs other than kidneys were examined<br>c) Studies where perfusion of the kidney is not isolated from the body (e.g. normothermic perfusion)<br>d) Studies where perfusion was not hypothermic perfusion (> 12°C) [3]<br>e) Studies not reporting on metabolism related outcome data or reporting on exogenous metabolites<br>4. No full text available

Table S3: Overview of study set-up and perfusion characteristics

Reference	Species	Temp (°C)	Severity injury	Perfusion time (h)	Perfusion Device	Pressure (mmHg)	Gas Type	FiO2 (%)	Gas Flow (L/min)	PO_2 (mmHg)
Alexander, 1970 [4]	Dog	8-10	Minimal	4, 8, 24	Home made (pulsatile pump, cooling unit, heat exchanger, silastic membrane oxygenator)	NA	O_2/CO_2	95%-99%	NA	500-600
Collste, 1971 [5]	Dog	8-10	Minimal/Injured	24-72	Home made (roller pump, thermostat; run-off oxygenator)	NA	NA	NA	NA	NA
Huang, 1971 [6]	Dog	10	Injured	24	Home made (pulsatile pump, heat exchanger, in-line filter, pressure manometer, membrane oxygenator)	60 systolic	O_2	NA	NA	130-140
Grundmann, 1972 [7]	Dog	3-4/8-10	Minimal	48-72	Belzer L1-400 (membrane oxygenator)	60 systolic	O_2/air	NA/21%	NA	130/50-500/180-250
Pegg, 1972 [8]	Rabbit	5	Minimal	24/48	Home made (membrane oxygenator)	60 (for 5 min) then 40	O_2/CO_2	95%	NA	650-700
Pedersen, 1973 [9]	Dog	10	Minimal	36	Gambro perfusion machine (Gambro PF 2A, membrane oxygenator)	60/40 (for 30 min) then 90/60	O_2/CO_2	No/98%	NA	75-146/270-475
Grundmann, 1974 [10]	Dog	6-7	Minimal	up to 120	Belzer L1-400 (membrane oxygenator)	40-45	NA	NA	NA	NA
Pettersson, 1974 [11]	Dog	5-7	Minimal	144	Gambro perfusion machine (Gambro PF 2A membrane oxygenator)	60 systolic	$O_2/CO_2/N_2$	33% CO_2, 1%; N_2, 66%	0.3	NA
Halasz, 1975 [12]	Dog	7	Minimal	72	Waters Medical systems Mox 100 cassette (membrane oxygenator)	45 mean	NA	NA	NA	NA
Lundstam, 1975 [13]	Dog + human	6-8	Minimal	144	Gambro perfusion machine (Gambro PF 2A membrane oxygenator)	60	$O_2/CO_2/N_2$	33% CO_2, 1%; N_2, 66%	0.3	NA
Lundstam, 1976 [14]	Dog	6-8	Minimal	144	Gambro perfusion machine (Gambro PF 2A membrane oxygenator)	60	$O_2/CO_2/N_2$	33% CO_2, 1%; N_2, 66%	0.3	NA
Slaattelid, 1976(1) [15]	Dog	8-10	Minimal	48	Gambro perfusion machine (Gambro PF 2A membrane oxygenator)	60 systolic	$O_2/CO_2/N_2$	33% CO_2, 1%; N_2, 66%	NA	NA

Reference	Species	Temp (°C)	Severity injury	Perfusion time (h)	Perfusion Device	Pressure (mmHg)	Gas Type	FiO2 (%)	Gas Flow (L/min)	PO_2 (mmHg)
Slaattelid, 1976(2) [16]	Dog	8-10	Minimal	48	Gambro perfusion machine (membrane oxygenator)	60 systolic	$O_2/CO_2/N_2$	33% CO_2, 1%; N_2, 66%	NA	NA
Collins, 1977 [17]	Dog	7	Minimal/injured	72	Waters Medical systems (MOX 100 cassette)	45 mean	NA	NA	NA	NA
Lundstam 1977(1) [18]	Dog	8-10	Minimal	144	Gambro perfusion machine (Gambro PF 2D surface oxygenation)	60	O_2/CO_2	99%	NA	NA
Lundstam, 1977(2) [19]	Dog	8-10	Minimal	144	Gambro perfusion machine (Gambro PF 2D surface oxygenation)	60	O_2/CO_2	99%	NA	NA
Skrede, 1979 [20]	Dog	8-10	Minimal	48	Gambro perfusion machine (Gambro PF 2A membrane oxygenation)	60 systolic	$O_2/CO_2/N_2$	33% CO_2, 1%; N_2, 66%	NA	NA
Fischer, 1979 [21]	Dog	6	Minimal	up to 120	Home made (pump: American Optical Co. Bedford Mass. (Mod. 16460); cryostat: Lauda UK 40 DL (surface oxygenation))	30 initial	O_2/O_2-air/air/N_2	NA	NA	169-624/±62/±29/<8
Fischer, 1980 [22]	Dog	6	Minimal	120	Home made (pump: American Optical Co. Bedford Mass. (Mod. 16460); cryostat: Lauda UK 40 DL (surface oxygenation))	30 initial	O_2	NA	NA	225-338
Pegg, 1981 [23]	Rabbit	10	Minimal/injured	24-48	Watson-Marlow type MHRE (membrane-, film oxygenator, surface oxygenation)	40	O_2/CO_2	95%	NA	150/650
Kleist, 1982 [24]	Dog + human	8-10	Minimal/injured	144	Gambro perfusion machine (Gambro PF 2D surface oxygenation)	60	O_2	99%	NA	NA
Kahng, 1983 [25]	Human	8	Minimal/Injured	26-36	NA	NA	NA	NA	NA	100
Pegg, 1984 [26]	Dog	10	Injured	48	Gambro perfusion machine (Gambro PF 3B membrane oxygenator)	NA	O_2/CO_2	95%	NA	590-620/240-260 with fluorocarbon
Southard, 1984(1) [27]	Dog	6-8	Minimal	24-120	Home made (pulsatile pump, surface oxygenation)	60 (at start) then 40-45 after 1-2 h	Air	21%	NA	150 ± 20

Reference	Species	Temp (°C)	Severity injury	Perfusion time (h)	Perfusion Device	Pressure (mmHg)	Gas Type	FiO2 (%)	Gas Flow (L/min)	PO_2 (mmHg)
Southard, 1984(2) [28]	Dog	6-8	Minimal	24-120	Home made (pulsatile pump, surface oxygenation)	60 (at start) then 40-45 after 1-2 h	Air	21%	NA	150 ± 20
Southard, 1984(3) [29]	Dog	6-10	Minimal	72-120	Home made (pulsatile pump, surface oxygenation)	60 (at start) then 30-40	O_2	100%	NA	300-400
Verkh, 1986 [30]	Dog	6 ± 2	Minimal	24	Waters Medical systems (MOX 100 cassette), (membrane oxygenator)	Initial pressure: 60, decrease to 40-45 within 1 h	O_2/CO_2	NA	1/0.05	300-350
McAnulty, 1988 [31]	Dog	6	Minimal	72-120	Home made (pulsatile pump, surface oxygenation)	Initial systolic pressure: 50	O_2	100%	3	NA
Boudjema, 1991 [32]	Dog	5	Minimal	120	Belzer L1-400, (surface oxygenation)	50	O_2	NA	5	100 ± 5
Baicu, 2004 [33]	Pig	5-7	Injured	24	LifePort Kidney Transporter	30-60	NA	NA	NA	NA
Minor, 2005 [34]	Pig	6-8	Injured	18	Home made (roller pump, pulsatile, tube oxygenator)	40/20 (at 50 cycles/min)	O_2	NA	NA	> 500
Baicu, 2006 [35]	Pig	5-8	Injured	72	LifePort Kidney Transporter	30-50	NA	NA	NA	NA
La Manna, 2009 [36]	Pig	4-7	Injured	15	Waters Medical systems (RM3)	45.6 ± 8.4	NA	NA	NA	NA
Buchs, 2011 [37]	Pig	2-4	Minimal/injured	18/8	Home made (membrane oxygenator)	50/15 max	O_2	NA	NA	750/375
Lazeyras, 2012 [38]	Pig	4	Minimal	8	Home made (membrane oxygenator, surface oxygenation)	45/15	O_2	NA	NA	750/375/75
Bon, 2014 [39]	Pig	4 ± 2	Injured	22	LifePort Kidney Transporter	35 (initial)	NA	NA	NA	NA
Nath, 2014 [40]	Pig + human	4	Injured	4	LifePort Kidney Transporter	30	NA	NA	NA	NA
Guy, 2015 [41]	Human	4	Injured	7-17	LifePort Kidney Transporter	30	NA	NA	NA	NA
Nath, 2016(1) [42]	Pig	4	Injured	24	LifePort Kidney Transporter	30	NA	NA	NA	NA
Nath, 2016(2) [43]	Pig	4	Injured	24	LifePort Kidney Transporter	30	NA	NA	NA	NA

Reference	Species	Temp (°C)	Severity injury	Perfusion time (h)	Perfusion Device	Pressure (mmHg)	Gas Type	FiO2 (%)	Gas Flow (L/min)	PO_2 (mmHg)
Hamaoui, 2016 [44]	Pig	7.8	Injured	10	Waters Medial systems RM3 perfusion machine	40 systolic	NA	NA	NA	> 100
Ravaioli, 2018 [45]	Human	4	Injured	3	Home made [Three peristaltic pumps (Medica S.P.A and Centro Iperbarico S.R.L) hyperbaric chamber, membrane oxygenator]	25-30	O_2/CO_2	NA	NA	No/750/750
Darius, 2018 [46]	Pig	3	Injured	± 22/2	LifePort Kidney Transporter, (membrane oxygenator)	30	O_2/CO_2 (95/5%)	30	NA	68/220-240/680-760
Kaminski, 2019 [47]	Pig	8.2 ± 1	Injured	20	LifePort Kidney Transporter	35	NA	NA	NA	145 at start, rapid decrease to 6.8
Patel, 2019 [48]	Pig	4	Injured	18	LifePort Kidney Transporter, (membrane oxygenator)	30	O_2/air	95%/21%	0.1/0.1	NA
Venema, 2019 [49]	Pig	4	Injured	24	Kidney Assist Tranporter, (membrane oxygenator)	25 mean	O_2	No/21%/ 100%	0.1	NA
Darius, 2020(1) [50]	Pig	2-8	Injured	22	LifePort Kidney Transporter, (membrane oxygenator)	30	O_2/CO_2 (95/5%)	30%	NA	68 ± 3/219 ± 13/717 ± 56/789 ± 14
Darius, 2020(2) [51]	Pig	2-8	Injured	± 22	LifePort Kidney Transporter, (membrane oxygenator)	30	O_2/CO_2 (95/5%)	NA	NA	70 ± 5/222 ± 8/718 ± 40
Darius, 2020(3) [52]	Pig	2-8	Injured	± 22	LifePort Kidney Transporter, (Membrane oxygenator, buble-surface oxygenation)	30	O_2/CO_2 (95/5%)	30%	No/0.5	68 ± 3/219 ± 13/400-500/717 ± 56
Longchamp, 2020 [53]	Pig	4	Minimal/Injured	22	Home made (membrane oxygenator)	45/15	O_2	NA	NA	750
Faucher, 2022 [54]	Human	2.2-5.8	Injured	13.9 (10-17)	LifePort Kidney Transporter	NA	NA	NA	NA	NA
Mrakic-Sposta, 2023 [55]	Pig	4	Injured	8	Waters Medical Systems (RM3)	20 to 35 (+2/8 min)	NA	NA	NA	NA

kPa and mbar were converted to mmHg where needed

NA, not available

Table S4: Detailed Risk of Bias Assessment using SYRCLE's tool for articles reporting on animal studies

	Reference	Alexander, 1970 [4]	Collste, 1971 [5]	Huang, 1971 [6]	Grundmann, 1972 [7]	Pegg, 1972 [8]	Pedersen, 1973 [9]	Grundmann, 1974 [10]	Pettersson, 1974 [11]	Halasz, 1975 [12]	Lundstam, 1975 [13]	Lundstam, 1976 [14]	Slaattelid (1), 1976 [15]	Slaattelid (2), 1976 [16]	Collins, 1977 [17]	Lundstam (1), 1977 [18]	Lundstam (2), 1977 [19]	Fischer, 1979 [21]
**Selection bias**	**1. Was the allocation sequence adequately generated and applied?**	?	?	-	?	?	?	?	?	?	-	?	?	?	?	-	?	?
	1a. Did the investigators describe a random component in the sequence generation process such as: (*)	?	?	-	?	?	?	?	?	?	-	?	?	?	?	-	?	?
	**2. Were the groups similar at baseline or were they adjusted for confounders in the analysis?**	+	+	?	+	+	+	+	+	+	-	+	+	+	?	-	+	+
	2a. Was the distribution of relevant baseline characteristics balanced for the intervention and control groups?	+	+	?	+	+	+	+	+	+	-	+	+	+	?	-	+	+
	2b. If relevant, did the investigators adequately adjust for unequal distribution of some relevant baseline characteristics in the analysis?	/	/	-	/	/	/	/	/	/	-	/	/	/	-	-	/	/
	2c. Was the timing of disease induction adequate?	?	?	?	?	?	?	?	?	?	?	?	?	?	?	?	?	?
	**3. Was the allocation to the different groups adequately concealed during?**	?	?	?	?	?	?	?	?	?	-	?	?	?	?	-	?	?
	3a. Could the investigator allocating the animals to intervention or control group not foresee assignment due to one of the following or equivalent methods?	?	?	?	?	?	?	?	?	?	-	?	?	?	?	?	?	?

	Reference	Alexander, 1970 [4]	Collste, 1971 [5]	Huang, 1971 [6]	Grundmann, 1972 [7]	Pegg, 1972 [8]	Pedersen, 1973 [9]	Grundmann, 1974 [10]	Pettersson, 1974 [11]	Halasz, 1975 [12]	Lundstam, 1975 [13]	Lundstam, 1976 [14]	Slaattelid (1), 1976 [15]	Slaattelid (2), 1976 [16]	Collins, 1977 [17]	Lundstam (1), 1977 [18]	Lundstam (2), 1977 [19]	Fischer, 1979 [21]
**Performance bias**	**4. Were the animals randomly housed during the experiment?**	?	?	?	?	?	?	?	?	?	?	?	?	?	?	?	?	?
	4a. Did the authors randomly place the cages or animals within the animal room/facility?	?	?	?	?	?	?	?	?	?	?	?	?	?	?	?	?	?
	4b. Is it unlikely that the outcome or the outcome measurement was influenced by not randomly housing the animals?	+	?	?	+	+	?	+	?	+	?	?	+	+	+	?	?	?
	**5. Were the caregivers and/or investigators blinded from knowledge which intervention each animal received during the experiment?**	?	?	?	?	?	?	?	?	?	?	?	?	?	?	?	?	?
	5a. Was blinding of caregivers and investigators ensured, and was it unlikely that their blinding could have been broken?	?	?	?	?	?	?	?	?	?	?	?	?	?	?	?	?	?
**Detection bias**	**6. Were animals selected at random for outcome assessment?**	?	?	?	?	?	?	?	?	?	?	?	?	?	?	-	?	?
	6a. Did the investigators randomly pick an animal during outcome assessment, or did they use a random component in the sequence generation for outcome assessment?	?	?	?	?	?	?	?	?	?	?	?	?	?	?	-	?	?
	**7. Was the outcome assessor blinded?**	?	?	?	?	?	?	?	?	?	?	?	?	?	?	?	?	?
	7a. Was blinding of the outcome assessor ensured, and was it unlikely that blinding could have been broken?	?	?	?	?	?	?	?	?	?	?	?	?	?	?	?	?	?

	Reference	Alexander, 1970 [4]	Collste, 1971 [5]	Huang, 1971 [6]	Grundmann, 1972 [7]	Pegg, 1972 [8]	Pedersen, 1973 [9]	Grundmann, 1974 [10]	Pettersson, 1974 [11]	Halasz, 1975 [12]	Lundstam, 1975 [13]	Lundstam, 1976 [14]	Slaattelid (1), 1976 [15]	Slaattelid (2), 1976 [16]	Collins, 1977 [17]	Lundstam (1), 1977 [18]	Lundstam (2), 1977 [19]	Fischer, 1979 [21]
	7b. Was the outcome assessor not blinded, but do review authors judge that the outcome is not likely to be influenced by lack of blinding?	/	/	/	/	/	/	/	/	/	/	/	/	/	/	/	/	/
**Attrition bias**	**8. Were incomplete outcome data adequately addressed? (*)**	?	?	-	?	?	?	?	?	?	-	-	-	-	-	-	-	?
	8a. Were all animals included in the analysis?	?	?	?	?	?	+	?	?	?	?	-	?	?	?	?	?	?
	8b. Were the reasons for missing outcome data unlikely to be related to true outcome? (e.g., technical failure)	?	?	?	?	?	?	?	?	?	?	?	?	?	?	?	?	?
	8c. Are missing outcome data balanced in numbers across intervention groups, with similar reasons for missing data across groups?	?	?	-	?	?	?	?	?	-	-	?	-	-	-	-	-	?
	8d. Are missing outcome data imputed using appropriate methods?	-	-	-	-	-	-	-	-	-	-	-	-	-	-	-	-	-
**Reporting bias**	**9. Are reports of the study free of selective outcome reporting? (*)**	?	?	-	-	?	?	-	+	-	-	-	-	-	-	-	-	?
	9a. Was the study protocol available and were all of the study's pre-specified primary and secondary outcomes reported in the current manuscript?	?	?	?	?	?	?	?	?	?	?	?	?	?	?	?	?	?
	9b. Was the study protocol not available, but was it clear that the published report included all expected outcomes (i.e. comparing methods and results section)?	?	?	-	-	?	?	-	+	-	-	-	-	-	-	-	-	?

	Reference	Alexander, 1970 [4]	Collste, 1971 [5]	Huang, 1971 [6]	Grundmann, 1972 [7]	Pegg, 1972 [8]	Pedersen, 1973 [9]	Grundmann, 1974 [10]	Pettersson, 1974 [11]	Halasz, 1975 [12]	Lundstam, 1975 [13]	Lundstam, 1976 [14]	Slaattelid (1), 1976 [15]	Slaattelid (2), 1976 [16]	Collins, 1977 [17]	Lundstam (1), 1977 [18]	Lundstam (2), 1977 [19]	Fischer, 1979 [21]
**Other bias**	**10. Was the study apparently free of other problems that could result in high risk of bias? (*)**	?	?	-	?	?	+	?	?	?	?	-	?	?	?	?	?	?
	10a. Was the study free of contamination (pooling drugs)?	-	-	-	-	-	-	-	-	+	-	-	-	-	-	-	-	-
	10b. Was the study free of inappropriate influence of funders?	?	?	?	?	?	?	?	?	?	?	?	?	?	?	?	?	?
	10c. Was the study free of unit of analysis errors?	?	?	-	?	?	+	?	?	?	?	-	?	?	?	?	?	?
	10d. Were design-specific risks of bias absent?	-	?	-	-	?	?	-	-	?	-	-	?	-	-	-	-	-
	10e. Were new animals added to the control and experimental groups to replace drop-outs from the original population?	?	?	?	?	?	/	?	?	?	?	?	?	?	?	?	?	?

+, yes = low risk; ?, unclear risk; -, no = high risk; / not relevant or applicable

Table S4 – Continued

	Reference	Skrede, 1979 [20]	Fischer, 1980 [22]	Pegg, 1981 [23]	Kleist, 1982 [24]	Pegg, 1984 [26]	Southard 1984(1) [27]	Southard 1984(2) [28]	Southard 1984(3) [29]	Verkh, 1986 [30]	McAnulty, 1988 [31]	Boudjema, 1991 [32]	Baicu, 2004 [33]	Minor, 2005 [34]	Baicu, 2006 [35]	La Manna, 2009 [36]	Buchs, 2011 [37]	Lazeyras, 2012 [38]	Bon, 2014 [39]
**Selection bias**	**1. Was the allocation sequence adequately generated and applied?**	?	?	?	-	?	-	-	-	-	?	?	?	?	?	?	?	?	?
	1a. Did the investigators describe a random component in the sequence generation process such as: (*)	?	?	?	-	?	-	-	-	-	?	?	?	?	?	?	?	?	?
	**2. Were the groups similar at baseline or were they adjusted for confounders in the analysis?**	-	+	+	-	+	?	?	?	-	?	+	+	+	+	+	+	+	+
	2a. Was the distribution of relevant baseline characteristics balanced for the intervention and control groups?	-	+	+	-	+	?	?	?	-	?	+	+	+	+	+	+	+	+
	2b. If relevant, did the investigators adequately adjust for unequal distribution of some relevant baseline characteristics in the analysis?	-	/	/	-	/	-	-	-	-	-	/	/	/	/	/	/	/	/
	2c. Was the timing of disease induction adequate?	?	?	?	?	?	?	?	?	?	?	?	?	?	?	?	?	?	?
	**3. Was the allocation to the different groups adequately concealed during?**	?	?	?	-	?	?	?	?	-	?	?	?	?	?	?	?	?	+
	3a. Could the investigator allocating the animals to intervention or control group not foresee assignment due to one of the following or equivalent methods?	?	?	?	-	?	?	?	?	-	?	?	?	?	?	?	?	?	?

	Reference	Skrede, 1979 [20]	Fischer, 1980 [22]	Pegg, 1981 [23]	Kleist, 1982 [24]	Pegg, 1984 [26]	Southard 1984(1) [27]	Southard 1984(2) [28]	Southard 1984(3) [29]	Verkh, 1986 [30]	McAnulty, 1988 [31]	Boudjema, 1991 [32]	Baicu, 2004 [33]	Minor, 2005 [34]	Baicu, 2006 [35]	La Manna, 2009 [36]	Buchs, 2011 [37]	Lazeyras, 2012 [38]	Bon, 2014 [39]
**Performance bias**	**4. Were the animals randomly housed during the experiment?**	?	?	?	?	?	?	?	?	?	?	?	?	?	?	?	?	?	?
	4a. Did the authors randomly place the cages or animals within the animal room/facility?	?	?	?	?	?	?	?	?	?	?	?	?	?	?	?	?	?	?
	4b. Is it unlikely that the outcome or the outcome measurement was influenced by not randomly housing the animals?	?	?	?	?	+	?	?	?	?	+	?	?	+	?	+	?	?	+
	**5. Were the caregivers and/or investigators blinded from knowledge which intervention each animal received during the experiment?**	?	?	?	?	?	?	?	?	-	?	?	?	?	?	?	?	?	+
	5a. Was blinding of caregivers and investigators ensured, and was it unlikely that their blinding could have been broken?	?	?	?	?	?	?	?	?	-	?	?	?	?	?	?	?	?	+
**Detection bias**	**6. Were animals selected at random for outcome assessment?**	?	?	?	?	?	?	?	?	-	?	?	?	?	?	?	?	?	?
	6a. Did the investigators randomly pick an animal during outcome assessment, or did they use a random component in the sequence generation for outcome assessment?	?	?	?	?	?	?	?	?	-	?	?	?	?	?	?	?	?	?
	**7. Was the outcome assessor blinded?**	?	?	?	?	?	?	?	?	?	?	?	?	?	?	?	?	?	+
	7a. Was blinding of the outcome assessor ensured, and was it unlikely that blinding could have been broken?	?	?	?	?	?	?	?	?	?	?	?	?	?	?	?	?	?	+

	Reference	Skrede, 1979 [20]	Fischer, 1980 [22]	Pegg, 1981 [23]	Kleist, 1982 [24]	Pegg, 1984 [26]	Southard 1984(1) [27]	Southard 1984(2) [28]	Southard 1984(3) [29]	Verkh, 1986 [30]	McAnulty, 1988 [31]	Boudjema, 1991 [32]	Baicu, 2004 [33]	Minor, 2005 [34]	Baicu, 2006 [35]	La Manna, 2009 [36]	Buchs, 2011 [37]	Lazeyras, 2012 [38]	Bon, 2014 [39]
	7b. Was the outcome assessor not blinded, but do review authors judge that the outcome is not likely to be influenced by lack of blinding?	/	/	/	/	/	/	/	/	/	/	/	/	/	/	/	/	/	/
**Attrition bias**	**8. Were incomplete outcome data adequately addressed? (*)**	-	?	?	-	?	-	-	-	?	-	-	?	?	?	+	-	+	?
	8a. Were all animals included in the analysis?	?	?	?	-	?	?	?	?	?	?	?	?	?	?	+	-	-	?
	8b. Were the reasons for missing outcome data unlikely to be related to true outcome? (e.g., technical failure)	?	?	?	?	?	?	?	?	?	?	?	?	?	?	?	?	?	?
	8c. Are missing outcome data balanced in numbers across intervention groups, with similar reasons for missing data across groups?	-	?	?	?	?	-	-	-	?	-	-	?	?	?	?	-	+	?
	8d. Are missing outcome data imputed using appropriate methods?	-	-	-	-	-	-	-	-	-	-	-	-	-	-	+	-	+	-
**Reporting bias**	**9. Are reports of the study free of selective outcome reporting? (*)**	?	?	?	-	?	-	-	-	?	?	-	?	?	?	+	?	?	+
	9a. Was the study protocol available and were all of the study's pre-specified primary and secondary outcomes reported in the current manuscript?	?	?	?	?	?	?	?	?	?	?	?	?	?	?	?	?	?	?
	9b. Was the study protocol not available, but was it clear that the published report included all expected outcomes (i.e. comparing methods and results section)?	?	?	?	-	?	-	-	-	?	?	-	?	?	?	+	?	?	+

	Reference	Skrede, 1979 [20]	Fischer, 1980 [22]	Pegg, 1981 [23]	Kleist, 1982 [24]	Pegg, 1984 [26]	Southard 1984(1) [27]	Southard 1984(2) [28]	Southard 1984(3) [29]	Verkh, 1986 [30]	McAnulty, 1988 [31]	Boudjema, 1991 [32]	Baicu, 2004 [33]	Minor, 2005 [34]	Baicu, 2006 [35]	La Manna, 2009 [36]	Buchs, 2011 [37]	Lazeyras, 2012 [38]	Bon, 2014 [39]
**Other bias**	**10. Was the study apparently free of other problems that could result in high risk of bias? (*)**	?	?	?	-	?	-	-	-	?	-	-	+	?	+	?	?	+	+
	10a. Was the study free of contamination (pooling drugs)?	-	-	-	-	-	-	-	-	-	-	-	-	-	-	-	-	-	-
	10b. Was the study free of inappropriate influence of funders?	?	?	?	?	?	?	?	?	?	?	?	?	?	?	?	?	?	?
	10c. Was the study free of unit of analysis errors?	?	?	?	-	?	-	-	-	?	-	-	?	?	?	?	-	?	?
	10d. Were design-specific risks of bias absent?	-	?	?	-	?	-	-	-	-	-	-	?	?	?	?	?	?	?
	10e. Were new animals added to the control and experimental groups to replace drop-outs from the original population?	?	?	?	-	?	?	?	?	?	?	?	?	?	?	/	-	-	?

+, yes = low risk; ?, unclear risk; -, no = high risk; / not relevant or applicable

Table S4 – continued

	Reference	Nath, 2014 [40]	Hamaoui, 2016 [44]	Nath(1), 2016 [42]	Nath(2);2016 [43]	Darius, 2018 [46]	Patel, 2019 [48]	Kaminski, 2019 [47]	Venema, 2019 [49]	Darius 2020(1) [50]	Darius 2020(2) [51]	Darius 2020(3) [52]	Longchamp, 2020 [53]	Mrakic-Sposta, 2023 [55]
**Selection bias**	**1. Was the allocation sequence adequately generated and applied?**	?	?	?	+	+	+	?	?	+	+	?	?	+
	1a. Did the investigators describe a random component in the sequence generation process such as: (*)	?	?	?	+	+	+	?	?	+	+	?	?	+
	**2. Were the groups similar at baseline or were they adjusted for confounders in the analysis?**	-	+	+	+	+	+	+	+	+	+	+	?	+
	2a. Was the distribution of relevant baseline characteristics balanced for the intervention and control groups?	-	+	+	+	+	+	+	+	+	+	+	?	+
	2b. If relevant, did the investigators adequately adjust for unequal distribution of some relevant baseline characteristics in the analysis?	-	/	/	/	/	/	/	/	/	/	/	/	/
	2c. Was the timing of disease induction adequate?	?	?	?	?	?	?	?	?	?	?	?	?	?
	**3. Was the allocation to the different groups adequately concealed during?**	?	?	?	+	?	+	?	+	+	+	+	+	?
	3a. Could the investigator allocating the animals to intervention or control group not foresee assignment due to one of the following or equivalent methods?	?	?	?	+	?	+	?	?	?	?	?	?	?

	Reference	Nath, 2014 [40]	Hamaoui, 2016 [44]	Nath(1), 2016 [42]	Nath(2);2016 [43]	Darius, 2018 [46]	Patel, 2019 [48]	Kaminski, 2019 [47]	Venema, 2019 [49]	Darius 2020(1) [50]	Darius 2020(2) [51]	Darius 2020(3) [52]	Longchamp, 2020 [53]	Mrakic-Sposta, 2023 [55]
Performance bias	**4. Were the animals randomly housed during the experiment?**	?	?	?	?	?	+	?	?	?	?	?	?	?
	4a. Did the authors randomly place the cages or animals within the animal room/facility?	?	?	?	?	?	+	?	?	?	?	?	?	?
	4b. Is it unlikely that the outcome or the outcome measurement was influenced by not randomly housing the animals?	?	?	?	?	+	?	+	?	+	+	+	?	+
	**5. Were the caregivers and/or investigators blinded from knowledge which intervention each animal received during the experiment?**	?	?	?	+	?	+	?	+	+	+	+	+	?
	5a. Was blinding of caregivers and investigators ensured, and was it unlikely that their blinding could have been broken?	?	?	?	+	?	+	?	+	+	+	+	+	?
Detection bias	**6. Were animals selected at random for outcome assessment?**	?	?	?	+	+	+	?	?	+	+	?	?	+
	6a. Did the investigators randomly pick an animal during outcome assessment, or did they use a random component in the sequence generation for outcome assessment?	?	?	?	+	+	+	?	?	+	+	?	?	+
	**7. Was the outcome assessor blinded?**	?	?	?	+	?	+	?	+	+	+	+	+	?
	7a. Was blinding of the outcome assessor ensured, and was it unlikely that blinding could have been broken?	?	?	?	+	?	+	?	+	+	+	+	+	?

	Reference	Nath, 2014 [40]	Hamaoui, 2016 [44]	Nath(1), 2016 [42]	Nath(2);2016 [43]	Darius, 2018 [46]	Patel, 2019 [48]	Kaminski, 2019 [47]	Venema, 2019 [49]	Darius 2020(1) [50]	Darius 2020(2) [51]	Darius 2020(3) [52]	Longchamp, 2020 [53]	Mrakic-Sposta, 2023 [55]
	7b. Was the outcome assessor not blinded, but do review authors judge that the outcome is not likely to be influenced by lack of blinding?	/	/	/	/	/	/	/	/	/	/	/	/	/
**Attrition bias**	**8. Were incomplete outcome data adequately addressed? (*)**	?	+	?	?	+	?	+	?	+	+	+	?	+
	8a. Were all animals included in the analysis?	?	+	?	?	-	?	-	+	-	-	-	?	-
	8b. Were the reasons for missing outcome data unlikely to be related to true outcome? (e.g., technical failure)	?	-	?	?	?	?	?	?	?	?	?	?	?
	8c. Are missing outcome data balanced in numbers across intervention groups, with similar reasons for missing data across groups?	?	+	?	?	?	?	+	?	?	?	?	?	+
	8d. Are missing outcome data imputed using appropriate methods?	-	+	-	-	+	-	+	-	+	+	+	-	+
**Reporting bias**	**9. Are reports of the study free of selective outcome reporting? (*)**	?	+	+	+	+	+	+	+	+	+	+	?	+
	9a. Was the study protocol available and were all of the study's pre-specified primary and secondary outcomes reported in the current manuscript?	?	?	+	+	?	+	?	?	?	?	?	?	?
	9b. Was the study protocol not available, but was it clear that the published report included all expected outcomes (i.e. comparing methods and results section)?	?	+	/	/	+	/	+	+	+	+	+	?	+

	Reference	Nath, 2014 [40]	Hamaoui, 2016 [44]	Nath(1), 2016 [42]	Nath(2);2016 [43]	Darius, 2018 [46]	Patel, 2019 [48]	Kaminski, 2019 [47]	Venema, 2019 [49]	Darius 2020(1) [50]	Darius 2020(2) [51]	Darius 2020(3) [52]	Longchamp, 2020 [53]	Mrakic-Sposta, 2023 [55]
**Other bias**	**10. Was the study apparently free of other problems that could result in high risk of bias? (*)**	?	+	?	?	?	+	?	+	?	?	?	?	?
	10a. Was the study free of contamination (pooling drugs)?	-	-	-	-	-	?	-	-	-	-	-	-	-
	10b. Was the study free of inappropriate influence of funders?	?	?	?	?	?	?	?	?	?	?	?	?	?
	10c. Was the study free of unit of analysis errors?	-	?	-	-	?	?	?	?	?	?	?	?	?
	10d. Were design-specific risks of bias absent?	?	?	?	?	?	?	?	?	?	?	?	-	?
	10e. Were new animals added to the control and experimental groups to replace drop-outs from the original population?	?	/	?	?	-	?	-	/	-	-	-	?	-

+, yes = low risk; ?, unclear risk; -, no = high risk; / not relevant or applicable

Table S5: Quality assessment of studies including human kidneys according to the NIH quality assessment score

	Reference	Faucher, 2022 [54]	Guy, 2015 [41]	Kahng, 1983 [25]	Kleist, 1982 [24]	Lundstam, 1975 [13]	Nath, 2014 [40]	Ravaioli, 2018 [45]
**Described as randomized**	Was the study described as randomized, a randomized trial, a randomized clinical trial, or an RCT?	-	NA	-	NA	NA	NA	+
**Treatment allocation–two interrelated pieces**	Adequate randomization: was the method of randomization adequate (i.e., use of randomly generated assignment)?	NR	NA	NR	NA	NA	NA	NR
	Allocation concealment: was the treatment allocation concealed (so that assignments could not be predicted)?	NR	NA	NR	NA	NA	NA	NR
**Blinding**	Were study participants and providers blinded to treatment group assignment?	-	-	NR	-	-	-	NR
	Were the people assessing the outcomes blinded to the participants' group assignments?	NR	NR	NR	NR	NR	NR	NR
**Similarity of groups at baseline**	Were the groups similar at baseline on important characteristics that could affect outcomes (e.g., demographics, risk factors, co-morbid conditions)?	+	-	-	-	-	-	+
**Dropout**	Was the overall drop-out rate from the study at endpoint 20% or lower of the number allocated to treatment?	+	+	+	+	+	+	+
	Was the differential drop-out rate (between treatment groups) at endpoint 15 percentage points or lower?	+	NA	NA	NA	NA	NA	+
**Adherence**	Was there high adherence to the intervention protocols for each treatment group?	+	+	+	+	+	+	+
**Avoid other interventions**	Were other interventions avoided or similar in the groups (e.g., similar background treatments)?	+	+	+	+	+	+	+
**Outcome measures assessment**	Were outcomes assessed using valid and reliable measures, implemented consistently across all study participants?	+	+	-	+	+	+	+
**Power calculation**	Did the authors report that the sample size was sufficiently large to be able to detect a difference in the main outcome between groups with at least 80% power?	CD	NA	NR	NA	NA	NA	NR
**Prespecified outcomes**	Were outcomes reported or subgroups analyzed prespecified (i.e., identified before analyses were conducted)?	+	+	-	-	+	+	+
**Intention-to-treat analysis**	Were all randomized participants analyzed in the group to which they were originally assigned, i.e., did they use an intention-to-treat analysis?	+	NA	NA	NA	NA	NA	+

+, yes = good; ?, Other* = fair; -, no = poor

*Other: CD, cannot determine; NA, not applicable; NR, not reported

Table S6: Composition of different perfusion solutions studied in this review

Reference	Carbohydrates	Amino Acids/Proteins	Fatty Acids/Lipids	Others
Plasma based perfusate				
Alexander 1970 [4]	dextrose	cryoprecipitated, undiluted, microfiltered canine plasma; collected in acid-citrate-dextrose	total lipids; phospholipids; neutral lipids; lysolecithin; sphingomyelin; lecithin; phosphatidylethanolamine	sodium citrate; citric acid; insulin; hydrocortisone; penicillin; neomycin
Collste 1971 [5]	dextrose	cryoprecipitated, undiluted, microfiltered canine plasma; collected in acid-citrate-dextrose	total lipids; phospholipids; neutral lipids; lysolecithin; sphingomyelin; lecithin; phosphatidylethanolamine	sodium citrate; citric acid; insulin; hydrocortisone; penicillin
Huang 1971 [6]	dextrose; mannitol	cryoprecipitated, undiluted, microfiltered canine plasma; collected in acid-citrate-dextrose	sodium oleate	sodium citrate; citric acid; insulin; hydrocortisone; penicillin; [14C]-labeled oleate-albumin
Grundmann 1972 [7]	Glucose; dextrose	cryoprecipitated, undiluted, microfiltered canine plasma; collected in acid-citrate-dextrose	total lipids; phospholipids; neutral lipids; lysolecithin; sphingomyelin; lecithin; phosphatidylethanolamine	sodium citrate; citric acid; insulin; hydrocortisone; penicillin
Pedersen 1973 [9]	/	filtered human plasma	/	papaverine; penicillin
Grundmann 1974 [10]	dextrose	pooled canine plasma	/	prednisolone; penicillin
Kahng 1983 [25]	glucose	plasmanate: human protein fraction; human albumin; acetyltryptophan;	sodium caprylate	heparin; insulin; phenolsulfonphthalein; cephalothin; methylprednisolone
Pegg 1984 [26]	glucose; mannitol	protein fraction	caprylate (Octanoate); Total fat	hypoxanthine; ampicillin; hydrocortisone
Verkh 1986 [30]	dextrose	plasmanate: human protein fraction; human albumin; acetyltryptophan	sodium caprylate	insulin; penicillin
Albumin-based perfusates				
Pegg 1972 [8]	glucose; dextran 70	bovine serum albumin	/	papaverine; gentamycin sulphate
Grundmann 1974 [10]	dextrose	human albumin; total protein	/	penicillin; prednisolone; insulin
Pettersson 1974 [11]	glucose; [U-14C]glucose	human albumin	[1-14C] linoleate; [1-14C]palmitate; [1-14C]caprylate; [1-14C]myristic acid	insulin; papaverine; benzylpenicillin; hydrocortisone
Halasz 1975 [12]	/	human albumin (in Ringer's lactate)	non-esterified fatty acids; defatted albumin solution: no NEFA	lactate; phosphate buffer system
Lundstam 1975 [13]	[U-14C]glucose	human albumin	[14C] linoleate; [14C]palmitate; [14C]caprylate	/
Lundstam* 1976 [14]	glucose; [U-14C]glucose	bovine albumin (fraction V) "fatty acid-free"	fatty acid concentration: <0.05 mmol/L	insulin; papaverine; benzylpenicillin; hydrocortisone

Reference	Carbohydrates	Amino Acids/Proteins	Fatty Acids/Lipids	Others
Lundstam* 1976 [14]	glucose; [U-14C]glucose	human albumin - reduced fatty acids (charcoal treatment (Chen)[56])	reduced concentration of free fatty acids	insulin; papaverine; benzylpenicillin; hydrocortisone
Lundstam* 1976 [14]	glucose; [U-14C]glucose	human albumin	[14C]acetate	insulin; papaverine; benzylpenicillin; hydrocortisone; [14C]lactate
Slaattelid* 1976(1) [15]	glucose	human albumin - reduced fatty acids (charcoal treatment (Chen)[56])	reduced concentration of free fatty acids	insulin; papaverine; benzylpenicillin; hydrocortisone
Slaattelid* 1976(1) [15]	glucose	human albumin	[1-14C]palmitate	insulin; papaverine; benzylpenicillin; hydrocortisone
Slaattelid* 1976(2) [16]	glucose; [U-14C]glucose	human albumin	/	insulin; papaverine; penicillin; hydrocortisone
Slaattelid* 1976(2) [16]	mannitol	human albumin	/	insulin; papaverine; penicillin; hydrocortisone
Lundstam 1977(1) [18]	glucose	human albumin; L-[U-14C]leucine; L-[U-14C]threonine; aspartate; alanine; arginine; glycine; histidine; (iso)leucine; lysine; methionine; phenylalanine; proline; serine; threonine; tryptophan; valine; glutamine; tyrosine	/	insulin; papaverine; benzylpenicillin; hydrocortisone; puromycin hydrochloride
Lundstam 1977(2) [19]	glucose; [14C]glucose	human albumin; [14C]cycloleucine; aspartate; alanine; arginine; glycine; histidine; (iso)leucine; lysine; methionine; phenylalanine; proline; serine; threonine; tryptophan; valine; glutamine; tyrosine	[14C]caprylate	insulin; papaverine; benzylpenicillin; hydrocortisone
Collins 1977 [17]	dextrose; mannitol	salt-poor human serum albumin	/	insulin; penicillin; hydrocortisone
Skrede 1979 [20]	glucose	human albumin	[14C] linoleate; 14C]palmitate	insulin; papaverine; benzylpenicillin; hydrocortisone
Fischer 1979 [21]	glucose	human albumin	/	insulin; refobacin (gentamicin); prednisolone
Fischer 1980 [22]	glucose	human albumin	octanoate	N-acetyl-DL-tryptophanate; prednisolon; refobacin (gentamicin); procaine; insulin
Kleist 1982 [24]	glucose	human albumin aspartate; alanine; arginine; glycine; histidine; (iso)leucine; lysine; methionine; phenylalanine; proline; serine; threonine; tryptophan; valine; glutamine; tyrosine	/	insulin; papaverine; benzylpenicillin; hydrocortisone; DL-mevalonate; DL-[2-14C]mevalonate

Reference	Carbohydrates	Amino Acids/Proteins	Fatty Acids/Lipids	Others
Synthetic perfusates				
Fischer 1980 [22]	mannitol	Haemaccel	/	prednisolon; refobacin (gentamicin); procaine; insulin
Pegg 1981 [23]	glucose	Haemaccel	caprylate (octanoate); acetate	carbenicillin; hypoxanthine; pyruvate
Pegg 1984 [26]	glucose	Haemaccel	caprylate (octanoate)	carbenicillin; hypoxanthine; FC-43 fluorocarbon with pluronic-F68
Southard 1984(1) [27]	glucose; sodium/potassium and magnesium gluconate; HES	/	/	glutathione; HEPES; penicillin; dexamethasone; insulin
Southard 1984(2) [28]	glucose; sodium and magnesium gluconate; HES	/	/	glutathione; HEPES; penicillin; dexamethasone; insulin; adenosine; deoxycoformycin (flush)
Southard 1984(3) [29]	glucose; sodium gluconate; magnesium gluconate; HES	/	/	glutathione; HEPES; penicillin; dexamethasone; insulin; adenosine
McAnulty 1988 [31]	glucose; ribose; sodium and magnesium gluconate; raffinose; HES	/	/	glutathione; HEPES; dexamethasone; insulin; penicillin; adenosine; allopurinol; adenine
Boudjema 1991 [32]	glucose; ribose; sodium/potassium and magnesium gluconate; HES; raffinose	glutamate; cysteine; glycine; cystine (cysteine disulfide); Thioproline	/	adenine; adenosine; allopurinol; glutathione (reduced and oxidized form HEPES
Baicu 2004 [33]	glucose; mannitol; ribose; sodium and magnesium gluconate; HES	/	/	HEPES (buffer); (reduced) glutathione; adenine
Minor 2005 [34]	glucose; mannitol; ribose; sodium and magnesium gluconate; HES	/	/	HEPES (buffer); (reduced) glutathione; adenine
Minor 2005 [34]	Glucose, mannitol	histidine; tryptophan; taurine	/	potassium hydrogen 2-ketoglutarate; heparin; ampicillin
Baicu 2006 [35]	Glucose; mannitol; ribose; sodium and magnesium gluconate; HES; FDP	/	/	HEPES (buffer); (reduced) glutathione; adenine
Baicu 2006 [35]	Glucose; mannitol; sucrose; lactobionate; gluconate; Dextran 40kDa	/	/	HEPES; adenosine; glutathione (reduced form)
La Manna 2009 [36]	Belzer solution, not clear	Belzer solution, not clear	Belzer solution, not clear	Belzer solution, not clear
Buchs 2011 [37]	glucose; mannitol; ribose; sodium and magnesium gluconate; HES	/	/	HEPES (buffer); (reduced) glutathione; adenine
Lazeyras 2012 [38]	glucose; mannitol; ribose; sodium and magnesium gluconate; HES	/	/	HEPES (buffer); (reduced) glutathione; adenine; insulin
Bon 2014 [39]	glucose; mannitol; ribose; sodium and magnesium gluconate; HES	/	/	HEPES (buffer); (reduced) glutathione; adenine ; Melagatran

Reference	Carbohydrates	Amino Acids/Proteins	Fatty Acids/Lipids	Others
Nath 2014 [40]	glucose; mannitol; ribose; sodium and magnesium gluconate; HES	/	/	HEPES (buffer); (reduced) glutathione; adenine
Guy 2015 [41]	glucose; mannitol; ribose; sodium and magnesium gluconate; HES	/	/	HEPES (buffer); (reduced) glutathione; adenine
Nath 2016(1) [42]	glucose; mannitol; ribose; sodium and magnesium gluconate; HES	/	/	HEPES (buffer); (reduced) glutathione; adenine
Nath 2016(2) [43]	glucose; mannitol; ribose; sodium and magnesium gluconate; HES	/	/	HEPES (buffer); (reduced) glutathione; adenine
Hamaoui 2016 [44]	potassium lactobionate; raffinose; HES	/	/	adenosine; glutathione; allopurinol; insulin; penicillin; dexamethasone
Ravaioli 2018 [45]	mannitol; lactobionate	glutamate; histidine	/	glutathione
Darius 2018 [46]	glucose; mannitol; ribose; sodium and magnesium gluconate; HES	/	/	HEPES (buffer); (reduced) glutathione; adenine
Kaminski 2019 [47]	glucose; mannitol; ribose; sodium and magnesium gluconate; HES	/	/	HEPES (buffer); (reduced) glutathione; adenine
Patel 2019 [48]	glucose; [U-13C]glucose; mannitol; ribose; sodium and magnesium gluconate; HES	/	/	HEPES (buffer); (reduced) glutathione; adenine
Venema 2019 [49]	glucose; mannitol; ribose; sodium and magnesium gluconate; HES	/	/	HEPES (buffer); (reduced) glutathione; adenine
Darius 2020(1) [50]	glucose; mannitol; ribose; sodium and magnesium gluconate; HES	/	/	HEPES (buffer); (reduced) glutathione; adenine
Darius 2020(2) [51]	glucose; mannitol; ribose; sodium and magnesium gluconate; HES	/	/	HEPES (buffer); (reduced) glutathione; adenine
Darius 2020(3) [52]	glucose; mannitol; ribose; sodium and magnesium gluconate; HES	/	/	HEPES (buffer); (reduced) glutathione; adenine
Longchamp 2020 [53]	glucose; mannitol; ribose; sodium and magnesium gluconate; HES	/	/	HEPES (buffer); (reduced) glutathione; adenine
Faucher 2022 [54]	glucose; mannitol; ribose; sodium and magnesium gluconate; HES	/	/	HEPES (buffer); (reduced) glutathione; adenine
Mrakic-Sposta 2023 [55]	glucose; mannitol; ribose; sodium and magnesium gluconate; HES	/	/	HEPES (buffer); (reduced) glutathione; adenine

*, this paper reports on experimental groups with different perfusates and these are presented separately in this table; FDP, fructose-1,6 diphosphate; NA, not available

Table S7: Extended summary of studies reporting on kidney metabolism with plasma based perfusate

Reference	Severity injury	Warm ischemia	Cold ischemia	Oxygen	Findings
Carbohydrate metabolism					
Alexander 1970 [4]	Minimal	No	No	Yes	**Perfusate:** no change in lactate, increase in pyruvate<br>lactate/pyruvate ratio is elevated at 30 minutes and returns to normal afterwards
Grundmann 1972 [7]	Minimal	No	No	Yes	**Perfusate:** increase in dextrose (10%)<br>lactate/pyruvate ratio: decrease (first hours)-plateau-increase (after 24h)
Pedersen 1973 [9]	Minimal	No	No	Yes/No	**Perfusate:** no change in glucose (similar with/without oxygenation),<br>decrease in lactate (greater decrease with oxygenator)
Grundmann1974 [10]	Minimal	No	No	Yes	**Perfusate:** increase in lactate/pyruvate ratio
Kahng 1983 [25]	Injured	3-6 min	0-10 h	Yes	**Tissue:** lactate highest measured metabolite (lactate/pyruvate ratio +/- 70)
Pegg 1984 [26]	Minimal/ Injured	60 min in Injured	No	Yes	**Perfusate:** decrease in glucose; increase in lactate; increase in pyruvate
Verkh 1986 [30]	Minimal	No	No	Yes	**Perfusate:** decrease in glucose; increase in lactate; increase in pyruvate;<br>lactate/pyruvate ratio increased by 11.3%
Amino Acid metabolism					
Verkh 1986 [30]	Minimal	No	No	Yes	**Perfusate:** increase in the concentration of almost all AA:<br>Statistically significant: alanine, glutamate, serine, glycine, valine, threonine, (iso)leucine, methionine, aspartate, phenylalanine, lysine (significant changes),<br>Statistically non-significant: proline, hydroxyproline, tyrosine, arginine, histidine (no significant change)<br>Two patterns: initial increase over the first 12hr and then plateau (alanine, serine, glutamate); continual increase over 24hr (phenylalanine, threonine, methionine)
**Fatty Acid metabolism**					
Huang 1971 [6]	Minimal	No	8-10 min	Yes	**Perfusate:** without oleate: 25% decrease in lipids (mainly triglycerides and relatively small amounts of phospholipids)<br>with oleate: decrease in triglycerides was only half of that without added oleate<br>**tissue:** without oleate: 35% decrease in total lipid (neutral lipids and triglycerides decreased most, total phospholipids decreased by only 27%)<br>with oleate: phospholipids decreased by only 8%
Grundmann 1972 [7]	Minimal	No	No	Yes	**Perfusate:** decrease in unesterified FA
Energy metabolism					
Collste 1971 [5]	Minimal/ Injured	20 min in Injured	No	Yes	**Tissue:** minimal injury: constant rise in ATP level<br>Injury: ATP decreased during warm ischemia and partial restoration of ATP with perfusion
Kahng 1983 [25]	Injured	3-6 min	0-10 h	Yes	**Tissue:** variable nucleotide contents with wide ranges in each nucleotide (Energy charge (ATP+0.5(ADP/TAN) average here was 0.40 (0.85-0.9 is the optimum)
Pegg 1984 [26]	Injured	60 min	No	Yes	**Tissue:** no restoration of adenine nucleotide

AA, amino acids; FA, fatty acids; TAN, total adenine nucleotide (ATP + ADP + AMP)

Table S8: Extended summary of studies reporting on kidney metabolism with albumin based perfusate

Reference	Severity injury	Warm ischemia	Cold ischemia	Oxygen	Findings
Carbohydrate metabolism					
Pegg 1972 [8]	Minimal	No	No	Yes	**Perfusate:** no change in glucose level, no measurable pyruvate production, lactate levels remained below 0.5mM
Grundmann 1974 [10]	Minimal	No	No	Yes	**Perfusate:** increase in lactate/pyruvate ratio<br>Preservation could be extended by perfusate exchange prior to rise in lactate/pyruvate ratio
Pettersson 1974 [11]	Minimal	No	No	Yes	**Perfusate:** decrease in glucose<br>Increase in lactate and lactate/pyruvate ratio<br>**Label (perfusate):** glucose label mainly seen in lactate, some in glycogen and CO_2
Lundstam 1975 [13]	NA	NA	NA	Yes	**Perfusate:** decrease in glucose<br>Increase in lactate (lower in FA-free with higher glucose oxidation)<br>**Label (perfusate):** modest incorporation of glucose label in CO_2<br>Similar findings in human and dog
Slaattelid 1976(2)* [16]	Minimal (glucose free perfusate)	No	No	Yes	**Perfusate:** glucose accumulation if animal received glucose infusion before procurement<br>**Tissue:** decrease in glucose concentration, decrease in renal glycogen, higher glucose concentration when glucose infusion was given before procurement compared with no glucose infusion
Slaattelid 1976(2)* [16]	Minimal (glucose rich perfusate)	No	No	Yes	**Perfusate:** decrease in glucose<br>Increase in lactate<br>**Label (perfusate):** decrease in labeled glucose with modest (3%) metabolization to lactate and CO_2<br>**Tissue:** high concentration of glucose
Lundstam 1976* [14]	Minimal	No	No	Yes	**Perfusate:** decrease in glucose (more pronounced initial decrease)<br>Increase in lactate (lowest in FA free perfusate)<br>**Label (perfusate):** incorporation of glucose carbon into CO_2 (greater in FA-free perfusate and more pronounced the first 3 days),<br>decrease in labeled lactate (labeled lactate recovered in CO_2 and glucose)
Lundstam 1976* [14]	Minimal	No	No	Yes	**Perfusate:** constant decrease in glucose<br>Increase in lactate (most pronounced with acetate, FA-rich perfusate)<br>**Label (perfusate):** incorporation of glucose carbon into CO_2 (less in FA-rich perfusate)<br>decrease in labeled lactate (labeled lactate recovered in CO_2 and glucose)
Lundstam 1977(2)* [19]	Minimal	No	No	Yes	**Perfusate:** slight decrease in glucose; Increase in lactate first 4days (plateau or decrease hereafter)<br>**Label (perfusate):** decrease in specific activity glucose
Lundstam 1977(2)* [19]	Minimal	No	No	Yes	**Perfusate:** greater decrease in glucose<br>Continuous increase in lactate
Fischer 1979 [21]	Minimal	2 min	No	Yes	**Tissue:** decrease in glucose (non-significant)<br>no increase in lactate
Fischer 1980 [22]	Minimal	2 min	No	Yes	**Tissue:** significant decrease in glucose<br>Stable lactate levels up to 72h, increase thereafter

Reference	Severity injury	Warm ischemia	Cold ischemia	Oxygen	Findings
Kahng 1983 [25]	Injured	5-20 min	5-5.5h	Yes	**Tissue:** lactate highest measured metabolite (lactate/pyruvate ratio +/- 70)
Amino Acid metabolism					
Lundstam 1977(1) [18]	Minimal	No	No	Yes	**Perfusate:** exponential decrease in leucine, slight increase in threonine<br>**Label (perfusate):** decrease in specific activity leucine and threonine<br>**Label (tissue):** higher incorporation in protein for leucine than threonine, (10x) higher incorporation in CO_2 for leucine than threonine
Lundstam 1977(2)* [19]	Minimal	No	No	Yes	**Perfusate:** rapid decrease in glutamine, proline, glycine, aspartate, arginine, slower decrease in (iso)leucine, methionine, valine, decrease in serine<br>status quo for histidine, cystine, lysine, phenylalanine<br>increase for threonine, tyrosine, ornithine, taurine, alanine, ammonia and urea, increase in glutamate the first days with decrease between 4th and 6th day<br>balanced uptake and release of nitrogen
Lundstam 1977(2)* [19]	Minimal	No	No	Yes	**Perfusate:** increase in almost all AA and ammonia (most pronounced for alanine and taurine)<br>no increase for glutamine, proline, aspartate, cystine<br>increase of nitrogen
Fatty Acid metabolism					
Pettersson 1974 [11]	Minimal	No	No	Yes	**Perfusate:** decrease in FFA: (fast decrease in short-chain FA (caprylate (8:0), decrease after 2 days in middle-chain FA (lauric acid (12:0) and myristic acid (14:0)), slow decrease in long-chain FA (palmitate (16:0), oleic acid (18:1), linoleate (18:2) and stearic acid (18:0))<br>**Label (perfusate):** high incorporation of caprylate label in glucose and lactate<br>**Tissue:** concentrations of triglycerides and phospholipids remained unchanged and cholesterol concentration decreased after 6 days of perfusion<br>**Label (tissue):** caprylate was predominantly oxidized to CO_2 (until day 4), increased incorporation of myristic acid in CO_2 (from day 4), low incorporation of labeled palmitate into CO_2, labeled linoleic, palmitic and myristic acid incorporated predominantly into phospholipids and triglycerides
Lundstam 1975 [13]	NA	No	No	Yes	**Perfusate:** fast decrease in FA (fast decrease caprylate, slow decrease followed by fast decrease (after 2-4 days) for lauric and myristic acid and slow decrease in palmitate, oleic acid and linoleate)<br>**Label (perfusate):** fast incorporation of caprylate label in CO_2 (first 3 days), from day 4 faster incorporation of myristic acid label in CO_2, modest incorporation of label from long-chain FA in CO_2
Halasz 1975 [12]	Minimal	No	No	Yes	**Perfusate:** increase in NEFA (FFA)
Lundstam 1976* [14]	Minimal	No	No	Yes	**Tissue:** decrease in phospholipids
Lundstam 1976* [14]	Minimal	No	No	Yes	**Label (perfusate):** decrease in labeled acetate with incorporation in CO_2 (first 2-4 days), glucose and lactate<br>**Tissue:** no change in cholesterol
Slaattelid 1976(1)* [15]	Minimal	No	No	Yes	**Perfusate:** fast linear decrease in FFA<br>**Label (perfusate):** slow decrease in labeled palmitate
Slaattelid 1976(1)* [15]	Minimal	No	No	Yes	**Perfusate:** low FFA was remained

Reference	Severity injury	Warm ischemia	Cold ischemia	Oxygen	Findings
Slaattelid 1976(2) [16]	Minimal	No	No	Yes	**Perfusate** decrease in FFA
Lundstam 1977(2) [19]	Minimal	No	No	Yes	**Perfusate:** higher decrease in FA with AA<br>rapid decrease in caprylate (depleted day 3-4)<br>**Label (perfusate):** high incorporation of labeled caprylate in CO_2
Fischer 1979 [21]	Minimal	2 min	No	Yes	**Perfusate:** first 24h: decrease in octanoate (= caprylate) (preferential consumption of octanoate), no decrease in long-chain FFA (increase in palmitate (16:0) and oleate (18:01),no decrease in stearic (18:0), linoleic (18:2) acid)<br>after 24h: depletion of octanoate and decrease in long-chain FFA
Skrede 1979 [20]	Minimal	No	No	Yes	**Perfusate:** decrease in caprylate, increase in all long-chain FA and arachidonic acid<br>**Label (perfusate):** only traces of palmitate and linoleate label in CO_2<br>**Tissue:** 10% decrease in total phospholipids, decrease in all FA except arachidonic acid, no change in total cholesterol and triglycerides<br>**Label (tissue):** incorporation of palmitate or linoleate label in tissue lipids (higher in phospholipids than triglycerides)
Kleist 1982 [24]	Minimal	No	No	Yes	**Label (perfusate):** decrease in mevalonate label, only small amounts incorporate in CO_2<br>**Tissue:** no decrease in cholesterol when mevalonate added<br>**Label (tissue):** mevalonate label incorporation in total lipid fraction of kidney cortex, (80% recovered in non-saponifiable lipid fraction (cholesterol and cholesterol precursors: lanosterol, squalene)) and 20% in saponifiable lipid fraction (FA containing lipids)
Energy metabolism					
Collins 1977* [17]	Minimal	No	No	Yes	**Tissue:** no change in TAN levels during 72h perfusion
Collins 1977* [17]	Injured	15,30 and 60 min	No	Yes	**Tissue:** *normothermia*: decrease in ATP, ADP and TAN (first 15' increase in AMP, thereafter decrease)<br>*perfusion*: regeneration of ATP and rise in energy charge (ATP+ 1/2 ADP/TAN) to near normal within the first hour after 24h: no significant regeneration of TAN
Fischer 1980 [22]	Minimal	2 min	No	Yes	**Tissue:** decrease in TAN and ATP<br>energy charge (EC= ATP +1/2 ADP/TAN) was optimal in both groups, (EC was significantly lower in the group without glucose after 72h)
Kahng 1983 [25]	Injured	5-20 min	5-5.5h	Yes	**Tissue:** variable nucleotide contents with wide ranges in each nucleotide (EC (ATP+0.5(ADP/TAN) average here was 0.40 (0.85-0.9 is the optimum)

AA, amino acids; (F)FA, (free) fatty acids; NEFA, non-esterified fatty acids = FFA; TAN, total adenine nucleotides (ATP + ADP + AMP); EC, energy charge
*, this paper reports on experimental groups with different perfusates and these are presented separately in this table

Table S9: Extended summary of studies reporting on kidney metabolism with synthetic perfusate

Reference	Severity injury	Warm ischemia	Cold ischemia	Oxygen	Findings
Carbohydrate metabolism					
Fischer 1980 [22]	Minimal	2 min	No	Yes	**Tissue:** low glucose and slight decrease in glucose<br>Stable lactate levels (+/- zero) without increase
Pegg 1981 [23]	Minimal/ Injured	3.5, 15, 30, 60 min in injured	No	Yes	**Perfusate:** acetate and pyruvate reduced glucose utilization, caprylate increased glucose removal, less lactate accumulation with higher pO_2 and/or hypoxanthine, increase in lactate and pyruvate when caprylate added, with glucose, small amount of pyruvate, with glucose and caprylate accumulation of pyruvate
Pegg 1984 [26]	Injured	60 min	No	Yes	**Perfusate:** decrease in glucose (same as PPF(plasma))<br>increase in lactate (lower than PPF(plasma))<br>increase in pyruvate (than PPF(plasma))
Baicu 2004 [33]	Injured	120 min	+/- 60 min	No	**Perfusate:** no change in glucose<br>no change in pyruvate (constant low value)<br>**Interstitial fluid** (micro dialysis): increase in pyruvate (higher concentrations in FDP-treated kidneys)
Baicu 2006* [35]	Injured	< 3 min	+/- 60 min	No	**Perfusate:** no change in glucose (perfusate replacement every 24h)
Baicu 2006* [35]	Inured	< 3 min	+/- 60 min	No	**Perfusate:** decrease in glucose (perfusate replacement every 24h)
Bon 2014 [39]	Injured	60 min	No	No	**Perfusate:** increase in lactate
Nath 2014 [40]	Injured	Pig: max 14 min<br>Human: DBD	Pig: 2 h<br>Human: DBD	No	**Perfusate:** no change in glucose<br>increase in lactate<br>no change in mannitol and ribose<br>number and concentration change of metabolites in pig and human perfusates are comparable
Guy 2015 [41]	Injured	3 DCD, 23 DBD	6-11 h	No	**Perfusate:** increase in glucose<br>Increase in lactate<br>no change in mannitol and ribose
Nath 2016(1) [42]	Injured	Max 14 min	2 h	No	**Perfusate:** increase in lactate<br>**Label (perfusate):** increase in labeled lactate from labeled glucose<br>**Label (tissue):** labeled lactate present in kidney cortex
Nath 2016(2) [43]	Injured	Max 14 min	2 h	No	**Perfusate:** Increase in lactate<br>**Tissue:** lactate amount in HMP tissue was lower than T0 tissue **Total metabolite amount (tissue + perfusate):** increase in lactate
Hamaoui 2016 [44]	Injured	+/- 15 min (11-20 min)	254 min 160-380 min	NA	**Perfusate:** increase in glucose levels (first 3h), decrease thereafter<br>increase in lactate<br>**Micro dialysis:** increase in cortical lactate (after 1.5h)<br>(medullary lactate levels were higher than cortical levels)
Ravaioli 2018 [45]	Injured	DBD	>20 h	No/Yes	**Perfusate:** increase in lactate
Darius 2018 [46]	Injured	30 min	No	Yes/No	**Perfusate:** increase in glucose (no difference oxygenated vs non-oxygenated)
Patel 2019 [48]	Injured	15 min	2 h	Yes	**Label (perfusate):** increase in labeled lactate from labeled glucose (reduction in increase in HMP02 vs steady increase in HMPair)<br>**Tissue:** lower cortical concentrations of labeled lactate in $HMPO_2$ vs HMPair.

Reference	Severity injury	Warm ischemia	Cold ischemia	Oxygen	Findings
Darius 2020(1) [50]	Injured	30 min	No	Yes	**Perfusate:** increase in lactate (in groups with pre-or end-oxygenation)<br>Increase in mannitol (in groups with pre-or end-oxygenation)<br>(all oxygenation strategies resulted in lower concentrations of lactate)<br>no differences in perfusate glucose between oxygen conditions<br>**Tissue**: no change in lactate
Darius 2020(2) [51]	Injured	30 min	No	Yes	**Perfusate:** increase in glucose (independent of oxygen content)<br>Lower lactate in HMPO_2high vs HMP<br>**Tissue:** increase in lactate
Darius 2020(3) [52]	Injured	30 min	No	Yes	**Perfusate:** increase in glucose (independent of oxygen content)<br>**Tissue:** no change in lactate
Faucher 2022 [54]	Injured	DBD	DBD	No	**Perfusate:** no change in glucose and mannitol<br>increase in lactate<br>decrease in ribose
Mrakic-Sposta 2023 [55]	Injured	75 min	No	No	**Perfusate:** increase in lactate<br>**Tissue:** increase in lactate
Amino Acid metabolism					
Boudjema 1991 [32]	Minimal	No	No	Yes	**Perfusate:** reduced glutathione disappeared from the perfusate in 24h (during 5 day perfusion, no trace of glutathione was detectable)<br>**Tissue:** loss of glutathione from the cortex tissue, perfusion with reduced glutathione suppressed this loss , perfusion with oxidized glutathione did not prevent this loss. Addition of glycine, glutamate and cysteine stimulated the synthesis of glutathione
Baicu 2004 [33]	Injured	120 min	+/- 60 min	No	**Perfusate:** increase in glutamate
Baicu 2006* [35]	Injured	< 3 min	+/- 60 min	No	**Perfusate:** increase in glutamate and ammonia (the first hours)
Baicu 2006* [35]	Injured	< 3 min	+/- 60 min	No	**Perfusate:** Baseline glutamine detected<br>Increase in glutamate (higher in UHK than Belzer MPS)<br>Increase in NH4+ (higher in UHK than Belzer MPS)
Bon 2014 [39]	Injured	60 min	No	No	**Perfusate:** increase in valine, alanine, glycine and glutamate
Nath 2014 [40]	Injured	Pig: max 14 min<br>Human: DBD	Pig: 2 h<br>Human: DBD	No	**Perfusate:** increase in glycine, glutamate, alanine, (iso)leucine and valine<br>no change in tyrosine<br>decrease in glutathione
Guy 2015 [41]	Injured	3 DCD, 23 DBD	6-11 h	No	**Perfusate:** increase in alanine, glycine, glutamate, (iso)leucine, tyrosine and valine<br>**Perfusate:** decrease in reduced glutathione<br>oxidized glutathione not detected
Nath 2016(1) [42]	Injured	Max 14 min	2 h	No	**Perfusate:** increase in alanine<br>**Label (perfusate):** increase in labeled alanine from labeled glucose<br>**Label (tissue):** labeled alanine from glucose present in kidney cortex, labeled(4,5 13C) glutamate in small amounts (<0.5% of total glutamate)

Reference	Severity injury	Warm ischemia	Cold ischemia	Oxygen	Findings
Nath 2016(2) [43]	Injured	Max 14 min	2 h	No	**Perfusate:** increase in alanine and glutamate<br>decrease in reduced glutathione<br>**Total metabolite amount (tissue + perfusate):** increase in glutamate, alanine, aspartate, leucine, tyrosine<br>**Tissue:** Absence of reduced glutathione
Patel 2019 [48]	Injured	15 min	2 h	Yes	**Perfusate:** alanine (increase HMPair-unchanged $HMPO_2$), aspartate: (unchanged HMPair, increase $HMPO_2$), glutamate (unchanged HMPair, decrease $HMPO_2$), glycine (unchanged HMPair and $HMPO_2$)<br>decrease in glutathione (in both oxygenation conditions)<br>**Label (perfusate):** increase in labeled alanine (reduction in increase in $HMPO_2$ vs steady increase in HMPair)<br>**Tissue:** higher concentrations of aspartate, tyrosine, valine, (glycine, alanine) in the cortex of $HMPO_2$ kidneys vs HMPair, lower glutamate in the cortex of $HMPO_2$ kidneys vs HMPair<br>**Label (tissue):** higher concentrations of [4,5-13C]glutamate in cortex of $HMPO_2$ vs HMP air<br>**Tissue:** higher cortex glutathione concentration in $HMPO_2$ vs HMPair
Darius 2020(1) [50]	Injured	30 min	No	Yes	**Perfusate:** increase in alanine, aspartate, glycine, (iso)leucine and glutamate (in groups with pre or end oxygenation)<br>decrease in glutathione (in groups with pre or end oxygenation (others are ancient groups, only end concentrations given)<br>**Tissue:** decrease in glutamate (glutamate levels were lower in oxygenated groups)
Darius 2020(2) [51]	Injured	30 min	No	Yes	**Perfusate:** glutathione (no difference between oxygen conditions**)**<br>**tissue:** no change in glutamate (glutamate levels were lower in oxygenated groups)
Darius 2020(3) [52]	Injured	30 min	No	Yes	**Tissue:** decrease in glutamate (glutamate levels were lower when oxygen was added for 2 h by membrane oxygenator than with bubble and intermittent surface oxygenation or when no O_2 was given)
Faucher 2022 [54]	Injured	DBD	DBD	No	**Perfusate:** Increase in tryptophan, de novo appearance in MPS: aspartate, serine, glycine, threonine, glutamate, alanine, (ornithine), proline, lysine, histidine, arginine, valine, methionine, tyrosine, (iso)leucine, phenylalanine, taurine<br>increase in AA correlated with perfusion duration<br>no change in glutathione (reduced form from MPS), decrease in oxidized glutathione
Mrakic-Sposta 2023 [55]	Injured	75 min	No	No	**Perfusate:** increase in valine and alanine<br>decrease in total glutathione levels<br>**Tissue:** increase in valine and alanine
Fatty Acid metabolism					
Southard 1984(1) [27]	Minimal	No	No	Yes	**Tissue:** decrease in phospholipids (first 24h) thereafter increase (no change in phosphatidylserine (PS)(10% of total phospholipids), initial decrease and increase to normal levels thereafter in phosphatidylethanolamine (PE)(31%), decrease in phosphatidylcholine after day 1, no changes afterwards for phosphatidylcholine (PC)(57%))<br>**isolated mitochondria:** initial decrease in phospholipids with increase after 3 days (FFA show gradual increase, PS shows no change after 5 days of perfusion, both PC and PE decrease (10%) after 1 day of perfusion, no further decrease up to 3 days, between 3th and 5th day there is an apparent increase in PE and PC levels)

Reference	Severity injury	Warm ischemia	Cold ischemia	Oxygen	Findings
Nath 2014 [40]	Injured	Pig: max 14 min<br>Human: DBD	Pig: 2 h<br>Human: DBD	No	**Perfusate:** no change in acetate (human + pig)<br>increase (human), no change (pig) in 3-hydroxybutyrate
Guy 2015 [41]	Injured	3 DCD, 23 DBD	6-11 h	No	**Perfusate:** no change in acetate<br>increase in ketone body 3-hydroxybutyrate
Nath 2016(1) [42]	Injured	Max 14 min	2 h	No	**Perfusate:** no change in acetate<br>**Label (perfusate):** no change in labeled acetate from labeled glucose (concentration = 1.25% at all time points)
Nath 2016(2) [43]	Injured	Max 14 min	2 h	No	**Total metabolite amount (tissue + perfusate):** increase in acetate
Patel 2019 [48]	Injured	15 min	2 h	Yes	**Perfusate:** decrease (HMPair) no change ($HMPO_2$) in acetate<br>**Tissue:** higher cortical concentration of acetate in $HMPO_2$
Darius 2020(1) [50]	Injured	30 min	No	Yes	**Perfusate:** decrease in acetate (in groups with pre-or end-oxygenation)
Darius 2020(2) [51]	Injured	30 min	No	Yes	**Perfusate:** acetate (lower in oxygenated groups)
Mrakic-Sposta 2023 [55]	Injured	75 min	No	No	**Perfusate:** increase in acetate
Energy metabolism					
Fischer 1980 [22]	Minimal	2 min	No	Yes	**Tissue:** decrease in TAN and ATP<br>energy charge (EC= ATP +1/2 ADP/ATP + ADP + AMP) was optimal in both groups, (EC was significantly lower in the group without glucose after 72h)
Pegg 1981 [23]	Minimal/ Injured	3.5, 15, 30, 60 min in injured	No	Yes	**Tissue:** decrease in TAN, ATP, ATP/ADP ratio with WI (high-energy phosphate stores depleted when glucose was sole energy source and pO_2 150mmHg) When oxygen tension was 600mmHg and with glucose, caprylate, hypoxanthine added, 5'nucleotide adenine levels maintained close to normal values; with 60' WIT: total AN level was restored to normal (only the ATP/ADP ratio was depressed) after 48h of perfusion
Pegg 1984 [26]	Minimal/ Injured	60 min in Injured	No	Yes	**Tissue:** significant adenine nucleotide restoration
Southard 1984(2) [28]	Minimal	No	No	Yes	**Perfusate:** decrease in adenosine<br>**Tissue:** decrease in ATP (loss can be prevented by including both adenosine (10mM) and PO4 (25mM)
Southard 1984(3) [29]	Minimal	No	No	Yes	**Tissue:** higher ATP content in cortex tissue after 3 days perfusion than control concentrations (perfusion with adenosine and PO4), concentration of ATP in cortex tissue from 5-day perfused kidneys was less than that of the control
McAnulty 1988 [31]	Minimal	No	No	Yes	**Perfusate**: almost complete degradation of adenosine during 5d perfusion<br>increase in hypoxanthine and inosine concentration<br>only 10% loss of adenine (no large increase in concentrations of purine end products)<br>**Tissue:** higher ATP and TAN concentration in kidney cortical tissue in adenine/ribose-perfused kidneys than in adenosine-perfused kidneys after 5 days
Minor 2005* [34]	Injured	40 min	No	Yes	**Tissue:** improved energy status with oxygenated Belzer perfusion (2.43 +- 0.23 µmol ATP/g) vs HTK (1.18 +- 0.12 µmol ATP/g)

Reference	Severity injury	Warm ischemia	Cold ischemia	Oxygen	Findings
Minor 2005* [34]	Injured	40 min	No	Yes	**Tissue:** improved energy status with oxygenated Belzer perfusion (2.43 +- 0.23 µmol ATP/g) vs HTK (1.18 +- 0.12 µmol ATP/g)
La Manna 2009 [36]	Injured	10, 15, 30 min	No	NA	**Tissue:** decrease in ATP levels (% decrease in ATP content was significantly lower in the perfusion than in the cold storage group)
Buchs 2011 [37]	Minimal/ Injured	0, 30 min	0, 8, 18 h	Yes	**Tissue:** increase in ATP (only with oxygenated perfusion)<br>decrease in ATP with WI
Lazeyras 2012 [38]	Minimal/ Injured	0	No, 10h CS	Yes	**Tissue:** ATP during perfusion - ATP depletion during cold storage - almost complete recovery of ATP during cold perfusion<br>(presence of ATP, PME and Pi with a pO_2 of 100 kPa, presence of PME, Pi and a decrease in ATP with pO_2 of 50 kPa and PME and Pi without ATP detectable with 20kPa) ? ATP detection only with high oxygen concentration?
Nath 2014 [40]	Injured	Pig: max 14 min<br>Human: DBD	Pig: 2 h<br>Human: DBD	No	**Perfusate:** increase in hypoxanthine and inosine<br>no change in adenine
Guy 2015 [41]	Injured	3 DCD, 23 DBD	6-11 h	No	**Perfusate:** increase in hypoxanthine and inosine<br>no change in adenine
	Injured	Max 14 min	2 h	No	**Total metabolite amount (tissue + perfusate):** increase in hypoxanthine<br>decrease in inosine
Ravaioli 2018 [45]	Injured	DBD	>20 h	No/Yes	**Tissue:** decrease in ATP (non-oxygenated perfusion)<br>increase in ATP (oxygenated and hyperbaric perfusion)<br>ATP was higher in the oxygenated and hyperbaric perfusion vs non-oxygenated perfusion, there is correlation between ATP and pCO2
Patel 2019 [48]	Injured	15 min	2 h	Yes	**Tissue:** higher levels of ATP, ADP in cortex in $HMPO_2$ vs HMPair<br>AMP comparable between $HMPO_2$ vs HMPair<br>no difference in adenosine concentration between $HMPO_2$ and HMPair
Kaminski 2019 [47]	Injured	60 min	No	No	**Tissue:** Increase in ATP with hypothermic perfusion after 60' WI decrease in ATP with 60' WI
Venema 2019 [49]	Injured	30 min	No	No/Yes	**Tissue:** no increase in ATP during 24h non-oxygenated perfusion<br>increase in ATP during 24h oxygenated (21% or 100%) perfusion<br>After 30' WI: ATP was almost completely depleted in every group
Darius 2020(1) [50]	Injured	30 min	No	Yes	**Perfusate:** increase in hypoxanthine<br>no change in adenine (in groups with pre or end oxygenation)<br>**Tissue:** increase in ATP and ADP level in pre-oxygenated group<br>decrease in AMP in all groups<br>higher ATP, ADP levels in pre-oxygenated group. (No differences in AMP, and NADH levels in oxygenated vs non-oxygenated groups)
Darius 2020(2) [51]	Injured	30 min	No	Yes	**Perfusate:** adenine, hypoxanthine(no difference between oxygen conditions)<br>**Tissue:** increase in ATP and ADP<br>no change in AMP

Reference	Severity injury	Warm ischemia	Cold ischemia	Oxygen	Findings
					(No differences in ATP, ADP and AMP with different oxygen concentrations)
Darius 2020(3) [52]	Injured	30 min	No	Yes	**Tissue:** increase in ATP, ADP in oxygenated groups<br>decrease in AMP<br>(ATP levels were higher in kidneys receiving oxygen vs no oxygen) No differences in ADP, AMP, and NADH levels in oxygenated vs non-oxygenated groups
Longchamp 2020 [53]	Minimal/ injured	0, 30, 60 min	No	Yes	**Tissue:** ATP increase (in the presence of oxygen)<br>0' WI: ATP remained stable up to 22h of perfusion; decrease in PME (PME containing AMP signal)<br>60' WI: decrease in total ATP but no change in PME
Faucher 2022 [54]	Injured	DBD	DBD	No	**Perfusate:** increase in inosine, xanthosine, xanthine, hypoxanthine and adenosine<br>decrease in adenine and ribose
TCA cycle metabolites					
Nath 2014 [40]	Injured	Pig: max 14 min<br>Human: DBD	Pig: 2 h<br>Human: DBD	No	**Perfusate:** increase in fumarate<br>no change in citrate
Guy 2015 [41]	Injured	3 DCD, 23 DBD	6-11 h	No	**Perfusate:** increase in citrate
Nath 2016(2) [43]	Injured	Max 14 min	2 h	No	**Total metabolite amount (tissue + perfusate):** increase in fumarate, succinate
Patel 2019 [48]	Injured	15 min	2 h	Yes	**Perfusate:** no change in fumarate (between oxygenation conditions)<br>**Tissue:** no difference in fumarate cortex concentration between oxygenation conditions<br>**Label (tissue):** Higher labeled succinate in cortex and medulla in $HMPO_2$ vs HMPair, labeling of citrate and malate in cortex and medulla
Darius 2020(1) [50]	Injured	30 min	No	Yes	**Perfusate:** no change in succinate (in groups with pre or end oxygenation<br>**Tissue:** decrease in succinate (in oxygenated group)s
Darius 2020(2) [51]	Injured	30 min	No	Yes	**Perfusate:** succinate (lower in $HMPO_2$)<br>**Tissue:** decrease in succinate (lower in oxygenated groups)
Darius 2020(3) [52]	Injured	30 min	No	Yes	**Tissue:** decrease in succinate (lower in 2h $HMPO_2$ group)
Faucher 2022 [54]	Injured	DBD	DBD	No	**Perfusate:** increase in alpha-keto-glutarate

AA, amino acids; FA, fatty acids; WI, warm ischemia; PPF, plasma protein fraction; HMP, hypothermic machine perfusion; MPS, machine perfusion solution (Belzer); TAN, total adenine nucleotide (ATP + ADP + AMP); FDP, fructose-1,6 diphosphate; T0, Time zero; UHK, perfusion solution (see table S6); EC, energy charge; PME, phosphomonoester (contains AMP peak); Pi, inorganic phosphate

*, this paper reports on experimental groups with different perfusates and these are presented separately in this table

References

1. Hooijmans, C.R.; Rovers, M.M.; de Vries, R.B.M.; Leenaars, M.; Ritskes-Hoitinga, M.; Langendam, M.W. SYRCLE's risk of bias tool for animal studies. *BMC Medical Research Methodology* **2014**, *14*, 43, doi:10.1186/1471-2288-14-43.
2. Study Quality Assessment Tools, NIH Available online: https://www.nhlbi.nih.gov/health-topics/study-quality-assessment-tools (accessed on 24/02/2023).
3. Karangwa, S.A.; Dutkowski, P.; Fontes, P.; Friend, P.J.; Guarrera, J.V.; Markmann, J.F.; Mergental, H.; Minor, T.; Quintini, C.; Selzner, M.; et al. Machine Perfusion of Donor Livers for Transplantation: A Proposal for Standardized Nomenclature and Reporting Guidelines. *Am J Transplant* **2016**, *16*, 2932-2942, doi:10.1111/ajt.13843.
4. Alexander, J.L.; Dmochowski, J.R.; Murray, J.E.; Couch, N.P. Successful 24 hour renal perfusion-preservation with monitoring by surface electrometry during the storage interval. *Surgery* **1970**, *67*, 944-950.
5. Collste, H.; Bergström, J.; Hultman, E.; Melin, B. ATP in the cortex of canine kidneys undergoing hypothermic storage. *Life Sciences* **1971**, *10*, 1201-1206, doi:10.1016/0024-3205(71)90289-x.
6. Huang, J.S.; Downes, G.L.; Belzer, F.O. Utilization of fatty acids in perfused hypothermic dog kidney. *J Lipid Res* **1971**, *12*, 622-627.
7. Grundmann, R.; Landes, T.; Pichlmaier, H. Hypothermic pulsatile perfusion of dog kidneys. Factors limiting successful preservation time. *Transplantation* **1972**, *14*, 742-747, doi:10.1097/00007890-197212000-00012.
8. Pegg, D.E.; Green, C.J. Renal preservation by hypothermic perfusion using a defined perfusion fluid. *Cryobiology* **1972**, *9*, 420-428, doi:10.1016/0011-2240(72)90159-9.
9. Pedersen, F.B.; Hrynczuk, J.R.; Scheibel, J.H.; Sorensen, B.L. Urine production and metabolism of glucose and lactic acid in the kidney during 36 hours of cooling and perfusion with diluted plasma. *Scand J Urol Nephrol* **1973**, *7*, 68-73, doi:10.3109/00365597309133675.
10. Grundmann, R.; Berr, F.; Pitschi, H. Ninety six hour preservation of canine kidneys. *Transplantation* **1974**, *17*, 299-305, doi:10.1097/00007890-197403000-00010.
11. Pettersson, S.; Claes, G.; Schersten, T. Fatty acid and glucose utilization during continuous hypothermic perfusion of dog kidney. *Eur Surg Res* **1974**, *6*, 79-94, doi:10.1159/000127708.
12. Halasz, N.A.; Collins, G.M. Fatty acid utilization during perfusion. *Transplantation* **1975**, *20*, 354-356.
13. Lundstam, S.; Claes, G.; Jonsson, O.; Naucler, J.; Pettersson, S.; Schersten, T. Metabolic studies on kidney in hypothermic perfusion. *Journal D Urologie Et De Nephrologie* **1975**, *81*, 716-720.
14. Lundstam, S.; Claes, G.; Jonsson, O.; Pettersson, S.; Schersten, T. Metabolism in the hypothermically perfused kidney. Production and utilization of lactate and utilization of acetate in the dog kidney. *Eur Surg Res* **1976**, *8*, 300-310, doi:10.1159/000127875.
15. Slaattelid, O.; Flatmark, A.; Skrede, S. The importance of perfusate content of free fatty acids for dog kidney preservation. *Scandinavian Journal of Clinical and Laboratory Investigation* **1976**, *36*, 239-245.
16. Slaattelid, O.; Gronnerod, O.; Horn, A. Preservation of dog kidneys by hypothermic perfusion with glucose-free and glucose-rich perfusates. *Eur Surg Res* **1976**, *8*, 515-527, doi:10.1159/000127898.
17. Collins, G.M.; Taft, P.; Green, R.D.; Ruprecht, R.; Halasz, N.A. Adenine nucleotide levels in preserved and ischemically injured canine kidneys. *World J Surg* **1977**, *2*, 237-243, doi:10.1007/bf01665093.
18. Lundstam, S.; Jagenburg, R.; Jonsson, O.; Lundholm, K.; Naucler, J.; Pettersson, S.; Schersten, T. Metabolism in the hypothermically perfused dog kidney. Incorporation rate of leucine and threonine into proteins. *Eur Surg Res* **1977**, *9*, 206-216, doi:10.1159/000127939.

19. Lundstam, S.; Jagenburg, R.; Jonsson, O.; Lundholm, K.; Naucler, J.; Pettersson, S.; Schersten, T. Metabolism in the hypothermically perfused dog kidney. Utilization and production of amino acids. *Eur Surg Res* **1977**, *9*, 191-205, doi:10.1159/000127938.
20. Skrede, S.; Slaattelid, O. Fatty acid metabolism during hypothermic perfusion of the isolated dog kidney. *Scand J Clin Lab Invest* **1979**, *39*, 765-771, doi:10.1080/00365517909108169.
21. Fischer, J.H.; Isselhard, W.; Hauer, U.; Menge, M. Free fatty acid and glucose metabolism during hypothermic perfusion of canine kidneys. *Eur Surg Res* **1979**, *11*, 107-121, doi:10.1159/000128057.
22. Fischer, J.H.; Armbruster, D.; Grebe, W.; Czerniak, A.; Isselhard, W. Effects of differences in substrate supply on the energy metabolism of hypothermically perfused canine kidneys. *Cryobiology* **1980**, *17*, 135-147, doi:10.1016/0011-2240(80)90018-8.
23. Pegg, D.E.; Wusteman, M.C.; Foreman, J. Metabolism of normal and ischemically injured rabbit kidneys during perfusion for 48 hours at 10 C. *Transplantation* **1981**, *32*, 437-443, doi:10.1097/00007890-198111000-00020.
24. Kleist, H.; Jonsson, O.; Lundstam, S.; Naucler, J.; Pettersson, S.; Schersten, T. Metabolism in the hypothermically perfused kidney: utilization of mevalonate in the human and in the dog kidney. *Eur Surg Res* **1982**, *14*, 236-244, doi:10.1159/000128294.
25. Kahng, M.W.; Trifillis, A.L.; Hall-Craggs, M.; Regec, A.; Trump, B.F. Biochemical and morphological studies on human kidneys preserved for transplantation. *Am J Clin Pathol* **1983**, *80*, 779-785, doi:10.1093/ajcp/80.6.779.
26. Pegg, D.E.; Foreman, J.; Rolles, K. Metabolism during preservation and viability of ischemically injured canine kidneys. *Transplantation* **1984**, *38*, 78-81.
27. Southard, J.H.; Ametani, M.S.; Lutz, M.F.; Belzer, F.O. Effects of hypothermic perfusion of kidneys on tissue and mitochondrial phospholipids. *Cryobiology* **1984**, *21*, 20-24, doi:10.1016/0011-2240(84)90018-x.
28. Southard, J.H.; Lutz, M.F.; Ametani, M.S.; Belzer, F.O. Stimulation of ATP synthesis in hypothermically perfused dog kidneys by adenosine and PO4. *Cryobiology* **1984**, *21*, 13-19, doi:10.1016/0011-2240(84)90017-8.
29. Southhard, J.H.; Kuniyoshi, M.; Lutz, M.F.; Ametani, M.; Belzer, F.O. Comparison of the effect of 3- and 5-day hypothermic perfusion of dog kidneys on metabolism of tissue slices. *Cryobiology* **1984**, *21*, 285-295, doi:10.1016/0011-2240(84)90324-9.
30. Verkh, L.; Freier, D.T.; Celik, C. Changes in concentration of amino acids and other metabolites during hypothermic perfusion of the canine kidney. *Cryobiology* **1986**, *23*, 366-370, doi:10.1016/0011-2240(86)90041-6.
31. McAnulty, J.F.; Southard, J.H.; Belzer, F.O. Comparison of the effects of adenine-ribose with adenosine for maintenance of ATP concentrations in 5-day hypothermically perfused dog kidneys. *Cryobiology* **1988**, *25*, 409-416, doi:10.1016/0011-2240(88)90048-x.
32. Boudjema, K.; Lindell, S.L.; Southard, J.H.; Belzer, F.O. Changes in glutathione concentration in hypothermically perfused dog kidneys. *J Lab Clin Med* **1991**, *117*, 131-137.
33. Baicu, S.C.; Simmons, P.M.; Campbell, L.H.; Taylor, M.J.; Brockbank, K.G. Interstitial fluid analysis for assessment of organ function. *Clin Transplant* **2004**, *18 Suppl 12*, 16-21, doi:10.1111/j.1399-0012.2004.00212.
34. Minor, T.; Sitzia, M.; Dombrowski, F. Kidney transplantation from non-heart-beating donors after oxygenated low-flow machine perfusion preservation with histidine-tryptophan-ketoglutarate solution. *Transpl Int* **2005**, *17*, 707-712, doi:10.1007/s00147-004-0795-3.
35. Baicu, S.C.; Taylor, M.J.; Brockbank, K.G. Modulating biochemical perturbations during 72-hour machine perfusion of kidneys: role of preservation solution. *Cryobiology* **2007**, *54*, 114-120, doi:10.1016/j.cryobiol.2006.11.001.
36. La Manna, G.; Conte, D.; Cappuccilli, M.L.; Nardo, B.; D'Addio, F.; Puviani, L.; Comai, G.; Bianchi, F.; Bertelli, R.; Lanci, N.; et al. An in vivo autotransplant model of renal preservation:

Cold storage versus machine perfusion in the prevention of ischemia/reperfusion injury. *Artificial Organs* **2009**, *33*, 565-570, doi:10.1111/j.1525-1594.2009.00743.x.
37. Buchs, J.B.; Lazeyras, F.; Ruttimann, R.; Nastasi, A.; Morel, P. Oxygenated hypothermic pulsatile perfusion versus cold static storage for kidneys from non heart-beating donors tested by in-line ATP resynthesis to establish a strategy of preservation. *Perfusion* **2011**, *26*, 159-165, doi:10.1177/0267659110387184.
38. Lazeyras, F.; Buhler, L.; Vallee, J.P.; Hergt, M.; Nastasi, A.; Ruttimann, R.; Morel, P.; Buchs, J.B. Detection of ATP by "in line" 31P magnetic resonance spectroscopy during oxygenated hypothermic pulsatile perfusion of pigs' kidneys. *Magnetic Resonance Materials in Physics, Biology and Medicine* **2012**, *25*, 391-399, doi:10.1007/s10334-012-0319-6.
39. Bon, D.; Billault, C.; Thuillier, R.; Hebrard, W.; Boildieu, N.; Celhay, O.; Irani, J.; Seguin, F.; Hauet, T. Analysis of perfusates during hypothermic machine perfusion by NMR spectroscopy: a potential tool for predicting kidney graft outcome. *Transplantation* **2014**, *97*, 810-816, doi:10.1097/TP.0000000000000046.
40. Nath, J.; Guy, A.; Smith, T.B.; Cobbold, M.; Inston, N.G.; Hodson, J.; Tennant, D.A.; Ludwig, C.; Ready, A.R. Metabolomic perfusate analysis during kidney machine perfusion: the pig provides an appropriate model for human studies. *PLoS One* **2014**, *9*, e114818, doi:10.1371/journal.pone.0114818.
41. Guy, A.J.; Nath, J.; Cobbold, M.; Ludwig, C.; Tennant, D.A.; Inston, N.G.; Ready, A.R. Metabolomic analysis of perfusate during hypothermic machine perfusion of human cadaveric kidneys. *Transplantation* **2015**, *99*, 754-759, doi:10.1097/TP.0000000000000398.
42. Nath, J.; Smith, T.; Hollis, A.; Ebbs, S.R.; Canbilen, S.W.; Tennant, D.A.; Ready, A.R.; Ludwig, C. 13 C glucose labelling studies using 2D NMR are a useful tool for determining ex vivo whole organ metabolism during hypothermic machine perfusion of kidneys. *Transplantation research* **2016**, *5*, 7-7, doi:10.1186/s13737-016-0037-0.
43. Nath, J.; Smith, T.B.; Patel, K.; Ebbs, S.R.; Hollis, A.; Tennant, D.A.; Ludwig, C.; Ready, A.R. Metabolic differences between cold stored and machine perfused porcine kidneys: A (1)H NMR based study. *Cryobiology* **2017**, *74*, 115-120, doi:10.1016/j.cryobiol.2016.11.006.
44. Hamaoui, K.; Gowers, S.; Damji, S.; Rogers, M.; Leong, C.L.; Hanna, G.; Darzi, A.; Boutelle, M.; Papalois, V. Rapid sampling microdialysis as a novel tool for parenchyma assessment during static cold storage and hypothermic machine perfusion in a translational ex vivo porcine kidney model. *J Surg Res* **2016**, *200*, 332-345, doi:10.1016/j.jss.2015.07.004.
45. Ravaioli, M.; Baldassare, M.; Vasuri, F.; Pasquinelli, G.; Laggetta, M.; Valente, S.; De Pace, V.; Neri, F.; Siniscalchi, A.; Zanfi, C.; et al. Strategies to Restore Adenosine Triphosphate (ATP) Level After More than 20 Hours of Cold Ischemia Time in Human Marginal Kidney Grafts. *Ann Transplant* **2018**, *23*, 34-44, doi:10.12659/aot.905406.
46. Darius, T.; Gianello, P.; Vergauwen, M.; Mourad, N.I.; Buemi, A.; De Meyer, M.; Mourad, M. The effect on early renal function of various dynamic preservation strategies in a preclinical pig ischemia–reperfusion autotransplant model. *American journal of transplantation : official journal of the American Society of Transplantation and the American Society of Transplant Surgeons* **2018**, *19*, 752-762, doi:10.1111/ajt.15100.
47. Kaminski, J.; Delpech, P.O.; Kaaki-Hosni, S.; Promeyrat, X.; Hauet, T.; Hannaert, P. Oxygen Consumption by Warm Ischemia-Injured Porcine Kidneys in Hypothermic Static and Machine Preservation. *J Surg Res* **2019**, *242*, 78-86, doi:10.1016/j.jss.2019.04.015.
48. Patel, K.; Smith, T.B.; Neil, D.A.H.; Thakker, A.; Tsuchiya, Y.; Higgs, E.B.; Hodges, N.J.; Ready, A.R.; Nath, J.; Ludwig, C. The Effects of Oxygenation on Ex Vivo Kidneys Undergoing Hypothermic Machine Perfusion. *Transplantation* **2019**, *103*, 314-322, doi:10.1097/TP.0000000000002542.
49. Venema, L.H.; Brat, A.; Moers, C.; t Hart, N.A.; Ploeg, R.J.; Hannaert, P.; Minor, T.; Leuvenink, A.; consortium, C. Effects of Oxygen During Long-term Hypothermic Machine Perfusion in a

Porcine Model of Kidney Donation After Circulatory Death. *Transplantation* **2019**, *103*, 2057-2064, doi:10.1097/TP.0000000000002728.
50. Darius, T.; Vergauwen, M.; Smith, T.; Gerin, I.; Joris, V.; Mueller, M.; Aydin, S.; Muller, X.; Schlegel, A.; Nath, J.; et al. Brief O(2) uploading during continuous hypothermic machine perfusion is simple yet effective oxygenation method to improve initial kidney function in a porcine autotransplant model. *Am J Transplant* **2020**, *20*, 2030-2043, doi:10.1111/ajt.15800.
51. Darius, T.; Vergauwen, M.; Smith, T.B.; Patel, K.; Craps, J.; Joris, V.; Aydin, S.; Ury, B.; Buemi, A.; De Meyer, M.; et al. Influence of Different Partial Pressures of Oxygen During Continuous Hypothermic Machine Perfusion in a Pig Kidney Ischemia-reperfusion Autotransplant Model. *Transplantation* **2020**, *104*, 731-743, doi:10.1097/TP.0000000000003051.
52. Darius, T.; Vergauwen, M.; Mueller, M.; Aydin, S.; Dutkowski, P.; Gianello, P.; Mourad, M. Brief Bubble and Intermittent Surface Oxygenation Is a Simple and Effective Alternative for Membrane Oxygenation During Hypothermic Machine Perfusion in Kidneys. *Transplant Direct* **2020**, *6*, e571, doi:10.1097/txd.0000000000001016.
53. Longchamp, A.; Klauser, A.; Songeon, J.; Agius, T.; Nastasi, A.; Ruttiman, R.; Moll, S.; Meier, R.P.H.; Buhler, L.; Corpataux, J.M.; et al. Ex Vivo Analysis of Kidney Graft Viability Using 31P Magnetic Resonance Imaging Spectroscopy. *Transplantation* **2020**, *104*, 1825-1831, doi:10.1097/TP.0000000000003323.
54. Faucher, Q.; Alarcan, H.; Sauvage, F.L.; Forestier, L.; Miquelestorena-Standley, E.; Nadal-Desbarats, L.; Arnion, H.; Venhard, J.C.; Brichart, N.; Bruyère, F.; et al. Perfusate Metabolomics Content and Expression of Tubular Transporters During Human Kidney Graft Preservation by Hypothermic Machine Perfusion. *Transplantation* **2022**, doi:10.1097/tp.0000000000004129.
55. Simona, M.-S.; Alessandra, V.; Emanuela, C.; Elena, T.; Michela, M.; Fulvia, G.; Vincenzo, S.; Ilaria, B.; Federica, M.; Eloisa, A.; et al. Evaluation of Oxidative Stress and Metabolic Profile in a Preclinical Kidney Transplantation Model According to Different Preservation Modalities. *International Journal of Molecular Sciences* **2023**, *24*, 1029-NA, doi:10.3390/ijms24021029.
56. Chen, R.F. Removal of Fatty Acids from Serum Albumin by Charcoal Treatment. *The Journal of biological chemistry* **1967**, *242*, 173-181, doi:10.1016/s0021-9258(19)81445-x.
